# Inorganic Nanoparticles as Radiosensitizers for Cancer Treatment

**DOI:** 10.3390/nano13212873

**Published:** 2023-10-30

**Authors:** Balaashwin Babu, Samantha Archer Stoltz, Agastya Mittal, Shreya Pawar, Elayaraja Kolanthai, Melanie Coathup, Sudipta Seal

**Affiliations:** 1 Advanced Materials Processing and Analysis Center, Department of Materials Science and Engineering, University of Central Florida, Orlando, FL 32826, USA; ashwin.babu@knights.ucf.edu (B.B.); samantha.stoltz@ucf.edu (S.A.S.); agastya.mittal@knights.ucf.edu (A.M.); shreya.pawar@knights.ucf.edu (S.P.); elayaraja.kolanthai@ucf.edu (E.K.); 2 Burnett School of Biomedical Sciences, College of Medicine, University of Central Florida, Orlando, FL 32827, USA; 3 Biionix Cluster, University of Central Florida, Orlando, FL 32827, USA; melanie.coathup@ucf.edu; 4 College of Medicine, University of Central Florida, Orlando, FL 32827, USA; 5 Nanoscience Technology Center, University of Central Florida, Orlando, FL, USA

**Keywords:** radiosensitizer, nanomaterials, reactive oxygen species, cancer, radiation therapy, cerium oxide, carbonaceous nanoparticles

## Abstract

Nanotechnology has expanded what can be achieved in our approach to cancer treatment. The ability to produce and engineer functional nanoparticle formulations to elicit higher incidences of tumor cell radiolysis has resulted in substantial improvements in cancer cell eradication while also permitting multi-modal biomedical functionalities. These radiosensitive nanomaterials utilize material characteristics, such as radio-blocking/absorbing high-Z atomic number elements, to mediate localized effects from therapeutic irradiation. These materials thereby allow subsequent scattered or emitted radiation to produce direct (e.g., damage to genetic materials) or indirect (e.g., protein oxidation, reactive oxygen species formation) damage to tumor cells. Using nanomaterials that activate under certain physiologic conditions, such as the tumor microenvironment, can selectively target tumor cells. These characteristics, combined with biological interactions that can target the tumor environment, allow for localized radio-sensitization while mitigating damage to healthy cells. This review explores the various nanomaterial formulations utilized in cancer radiosensitivity research. Emphasis on inorganic nanomaterials showcases the specific material characteristics that enable higher incidences of radiation while ensuring localized cancer targeting based on tumor microenvironment activation. The aim of this review is to guide future research in cancer radiosensitization using nanomaterial formulations and to detail common approaches to its treatment, as well as their relations to commonly implemented radiotherapy techniques.

## 1. Introduction

Cancer is a significant burden on modern human societies as life expectancies have improved. It is the second leading cause of death, with 21% of all global deaths attributed to this lethal illness [1]. While the mortality rates of cardiovascular diseases, the leading cause of global death, continue to decrease with targeted intervention and early detection, rates of cancer mortality are rising with the potential to overtake heart disease as the leading cause of death. This is apparent with cancer research now overtaking cardiovascular research as one of the top-researched and funded diseases [2,3]. According to the International Cancer Research Partnership (ICRP), nearly $80 billion dollars (USD) was contributed toward cancer research funding since 2000. Among the various categories of research, cancer treatment continues to be the largest investment category and continues to increase in absolute investment and percentage of the overall funding [4]. As can be summarized from the financial investments, a substantial amount of resources in all forms has been levied to better understand and address cancer evolution and progression.

While cancer treatment is a heavily funded venture, translation into clinically viable solutions is still a massive undertaking. This is due to the nature of this disease: with high rates of mutation and variation in character/progression from patient to patient, preventing a one-size-fits-all approach to treatment [5]. Causes for the switch from normal cell tissue to cancer cell tissue can be attributed to processes at the cellular level that include altered signaling and metabolism. However, the specific causes of this onset of cellular dysfunction are still unknown. Available data suggest a myriad of factors, including environmental, chemical, biological, physical, and genetic [6]. While these factors are being investigated to prevent and mitigate cancer, using these factors as a strategy plan for cancer elimination has been, as of yet, ineffective. In other words, the unknown cellular mechanisms that are responsible for cancer transformation are often still present even if strict medical and lifestyle interventions are administered.

Additionally, there are nearly 200 types of cancer, and each cancer type is classified by the origin of the disease within the body, as well as the type of cell that it originates from [7]. This classification results in major differences in the mortality rate of each cancer from upwards of 80% survival rate of certain melanomas to less than 10% survival rate of pancreatic cancer in a 5-year span [8]. A major contributor to the differing rates of mortality is the metastatic ability of each type of cancer, affected by a multitude of factors. Most significantly, the metastatic ability is affected by the immune environment, the cancer cell type origin, epigenetic factors, the metabolic profile of cancerous cells, etc. [9]. However, the adaptability of cancer to thrive in a myriad of environments within the human body creates a unique issue in therapeutic intervention. Cancer cells often survive despite radiation and chemotherapy treatments, which aim to eradicate cancer cells but also cause damage to normal cells. While certain chemotherapy drugs, such as inhibitors of mTOR, Mdm2, caspases, and mitogenic kinases, can protect normal cells from both chemotherapy and radiotherapy, innovative techniques can even reduce the overall dosage of radiation [10]. One such technique involves a reduction in radiation dosages by incorporating radiosensitizers. Radiosensitizers are defined as any chemical or pharmaceutical agent that increases or decreases the cytotoxicity of ionizing radiation [11]. Radiosensitizers work with radiation therapy to create a higher tumor inactivation [12].

**Figure 1:**
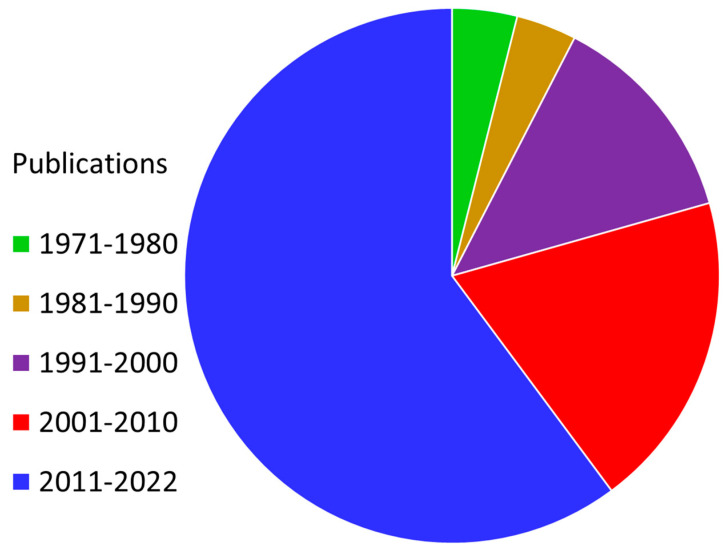
Prevalence of “radiosensitizer” as a keyword in the published research literature (1971–2022). Cancer is a widespread disease that has a significant negative impact on the modern world, with the number of affected individuals increasing daily. We are aware that it is currently incurable. There is a need for effective cancer treatments that do not disrupt the daily lives of individuals afflicted with the illness nor harm healthy cells outside of the scope of treatment. In this regard, radiation therapy stands out as one of the best therapeutic solutions for cancer treatment. This publication data were gathered from Web of Science, a platform provided by Clarivate Analytics.

Chemical radiosensitizers are often divided based on processes of DNA damage and repair [12]. The five categories include oxygen mimics, suppression of thiol groups, formation of toxic radiation products, structural incorporation of thymine analogs, and inhibition of post-irradiation processes [13]. While this classification system was critical at the time to engineer drug components to target specific cancer cell cycle arrests, new mechanisms and materials have been proposed that expand beyond this initial classification. Current research on radiosensitizers now categorizes these agents by their material characteristics, including divisions on macromolecules, small molecules, and nanomaterials [14] (Figure 1). While small molecules have already been extensively studied, macromolecules and nanomaterials are emerging as new frontiers in the world of radio-sensitization as their innovative mechanisms for enhanced cancer apoptosis create the possibility for new treatment regimens.

Macromolecules have the ability to regulate genetic expression to enhance radiation sensitivity for cancer cells. Certain genetic regulatory macromolecules such as protein, RNA, and DNA can either augment or mitigate sensitivity to radiation, leading to precision radiotherapy [15]. This revolution in cancer treatment utilizes the specific mechanisms responsible for each kind of cancer cell, as these processes are affected by radiation. However, the field of precision radiotherapy does not simply end with macromolecules, as chemical and structural characteristics are also utilized to effectively eliminate cancer cells. This is where the field of nanotechnology plays a key role.

Nanomaterials can combine macromolecules such as miRNAs and proteins with functional material characteristics [14,16]. These material characteristics include control of the radiation absorption and emission, higher selectivity, production of reactive oxidative species (ROS), up-conversion/down-conversion processes, and tumor microenvironment control [17,18,19]. While all these processes can be effective in killing tumor cells, the production of ROS can coincide with the processes of radiolysis of tumor cells. As radiation interacts with inorganic materials in the nanoscale, atoms are dislocated from their crystal lattice structure, resulting in vacancy-interstitial pairs [20,21]. These characteristics are important to consider when developing radiosensitizers and allow the nanotechnology-enabled radiosensitizers to be a new frontier in radiotherapy as well as cancer treatment in general. This review paper focuses on how the enhancement of radiotherapy through the use of various nanomaterials can contribute to eliminating cancer cells. This review paper evaluates the various approaches to radiosensitization as well as their association with clinically relevant radiotherapy techniques. An emphasis on research dating from 2000 to 2023 enables this review to showcase the recent developments in this emerging field of study. The various nanomaterials are investigated for their ability to act as radiosensitizers, survive within the tumor microenvironment, and be able to target cancer cells while keeping healthy cells intact.

This paper sets itself apart in that it explores the therapeutic potential of a wide array of inorganic materials, including metallics, semiconductors, and metal oxides. While organic nanosystems have shown promise in similar therapeutic mechanisms, their material character is markedly different from that of inorganics and, therefore, is not discussed in this review. The decision to focus this review on inorganic materials specifically is derived from the distinct structure, composition, and characterization of this category of nanosystem. Several recent papers report on the applications of nanotechnology in radio and photosensitization in general and with reference to oncological use [22,23,24,25,26,27]. These papers often either adopt a broad approach to the topic or discuss pinpointed applications. In isolating inorganics specifically, given the commonalities of this group of materials (i.e., lattice photons, band gaps, surface chemistry), we streamline the consistency of this paper and are able to provide more detailed explanations on a well-organized subset of potential therapies. We perceived this to be the ideal format for the readers’ ease of use in future implementations and research approaches.

Therefore, a number of valuable articles highlighting the efficacy of organic nanosystems in radio sensitization have recently surfaced [22,23,24,25,26,28,29]. Though other systems, such as liposomal or protein-based nanosystems, are currently being studied for similar use, we present a directed focus on the efficacy of inorganic nanoparticles (NPs) for radio and photosensitization due to their uniquely advantageous characteristics [22,28,30]. These notable merits include tailorable structure and function, enhanced radiotherapy outcomes, biocompatibility, precision in targeting, as well as potential complementary effects with thermal therapies [29,31,32,33]. While the outcome of inorganic and organic-based radiosensitivity is similar, there are discernable differences in operational mechanisms between the two. Organic systems tend to be less stable and have been known to undergo photooxidation or exhibit structural loss as a result of local heating. The biological environment can further contribute to the degradation of organic NPs, often resulting in unpredictable and toxic structures, especially under irradiation [34,35]. The typical mechanism of the inorganics we discuss in this paper is degradation into nontoxic minerals and metal ions, which tend to be more sustainable in the presence of radiation [36]. Further, we recognize the autocatalytic reactions of some inorganics, such as those that take place on the surface of cerium oxide NPs [37,38]. We also discuss the thermal properties of inorganic systems. Metals have the inherent ability to absorb radiation as well as exhibit property-dependent photon/vibration mode activation, as well as free-electron energy dissipation. Organic materials lack high-Z nuclei for scattering purposes, whereas inorganic substances are typically high-Z materials that possess highly absorptive lattice structures and charged lattice sites that facilitate scintillation and control the energies of photons through reflection, refraction, or diffraction to enhance local therapies [33].

## 2. Tumor Physiology

**Figure 2:**
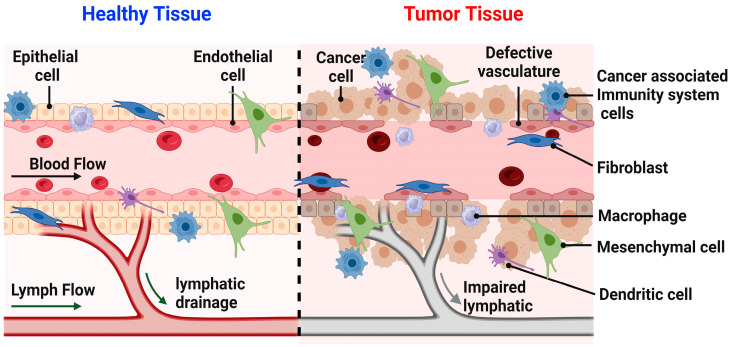
Illustrates the difference between the healthy and tumor tissues. In healthy tissue, various cell types such as epithelial cells, fibroblasts, macrophages, mesenchymal cells, dendritic cells, and immune response cells, lymphatic drainage is present. These components collectively represent the normal cellular functions within healthy tissue. Conversely, in tumor tissue, the visual depicts cancer cells alongside immune response cells, fibroblasts, macrophages, mesenchymal cells, and dendritic cells. Additionally, the illustration portrays compromised lymphatic drainage and abnormal vasculature. These elements collectively illustrate the irregular cellular functions characteristic of tumor tissue.

Knowledge of tumor physiology is crucial to pinpointing proper treatments for different cancers (Figure 2). The tumor environment contrasts greatly from that of healthy tissue and is generally characterized by abnormal structure and vasculature, heterogeneous hypoxic or anoxic conditions, low glucose and high lactate levels, increased permeability, and abnormal pH gradients [39]. These hostile characteristics of a tumor environment, among others, have a notable influence on treatment efficacy (Figure 2). For instance, it has been found that the hypoxic conditions of a tumor can drastically decrease its sensitivity to radiation therapy, often requiring doses up to three times higher than what would be needed in normal physiological conditions. This radioresistance of tumors has proven to be a huge obstacle to treatment and a notable cause of the inherent systemic side effects of these therapies [39].

Interactions between antioxidants and reactive oxygen species (ROS) are integral for the maintenance of biological processes, from homeostasis to tumorigenesis [40,41,42]. While ROS balance is required for body homeostasis, excess can result in oxidative stress, as well as related functional losses, DNA damage, or cell death [42]. ROS imbalances have long been associated with the cancer cycle, influencing tumorigenesis and progression, as well as apoptosis [43]. Though each type of cancer progresses differently in the presence of variable ROS types, the complicated relationship between ROS and cancer metastasis is noteworthy in the investigation of treatment mechanisms [44]. Luckily, the implementation of positron emission tomography (PET) has enhanced ROS measurement techniques for more accurate data on in vivo cancer models in recent years.

The human immune response can have an unexpected effect following carcinogenesis, often resulting in metastatic growth of a cancer [44]. Metastasis describes cancer cell dissemination toward organs or systems different from the parent tissue and results in 90% of solid tumor patient mortality [45]. Once cancer has reached metastasis, there is less confidence in the effectiveness of classical treatment options such as surgery or radiotherapy [46]. Since cancers that have metastasized are notoriously harder to approach with treatments than those confined to a single tissue type, inhibition of the cancer cell migration has been the topic of interest in many recent studies [47]. While previous attempts to employ antioxidants to halt ROS-assisted cancer progression and metastasis worked to little avail, current work assesses the efficacy of employing prooxidants to induce autophagy and apoptosis [48,49]. This seemingly contradictory strategy has shown some promise, with experimentations resulting in preliminary data that suggest nano-mediated ROS production results in targeted apoptosis of cancer cells [50,51].

Despite the increased hostility of the aforementioned tumor characteristics, scientists have been able to use knowledge of certain characteristics to their advantage. The tumor microenvironment (TME) consists of interactions between malignant and non-malignant cells (Figure 2). These non-malignant cells, such as those of the immune, vascular, lymphatic, and connective systems and their proteinaceous products, are often employed by the tumor for enhancement of growth and progression [46]. The vascular system of a tumor lacks supportive tissue, which results in leaky vessels and hyperpermeability. This, in addition to the lack of a properly functioning lymphatic system, means that molecules enter the tumor environment easily and are not readily evacuated [46].

Enhanced understanding of this EPR effect has incited excitement among the nanomedicine community. Nanotechnology has enhanced the delivery of drugs greatly due to the characteristically large surface, an important aspect to consider as it allows for both stabilization and protection of the cargo [52]. The nanotherapeutics in question are notable for their ability to provide sensitizing treatment to the cancer system. For example, targeted drug delivery with NPs can penetrate and remain in tumor tissue for continuous employment of the treatment mechanism. This synergy is often deemed “EPR-based tumor targeting” [53]. This site-specific nanomedicine can be used synergistically alongside classic treatments for cancer, namely, radiation therapy. Radiation can have amplified effects on a solid tumor with the help of radiosensitization [54]. For instance, certain metal NPs, namely, silver and gold, have been found to interact with the tumor tissue and increase the radiosensitivity [41,55].

Medical nanotechnology is the centerpiece of a group of emerging radiation treatment enhancements [56]. High atomic number elements are observed effectively absorbing radiation, acting as radio-sensitizers in a tumor environment, making these treatments more effective at a lower dose [56]. Nanomaterials can enhance the effectiveness of radiotherapy in multiple ways. While these nanosystems can increase the absorption of radiation based on their makeup, they can also prime the tissue to better receive the treatment by influencing the tumor environment. For example, these nanomedicines can combat the hypoxic conditions of the tissue or induce ROS production and make the tumor more sensitive to radiation [57]. Another notable synergy of these nanomaterials with radiotherapy is their radioprotective abilities. Certain nanomaterials may act as a buffer from harm to the healthy tissue surrounding a tumor receiving treatment [57]. With this greater understanding of both tumor characteristics and the latest therapeutic foci, nanomedicine has emerged with promise as a new centerpiece for cancer research and treatment. However, to understand the role of nanomedicine in clinically relevant outcomes, it is important to understand the current approaches to cancer treatment. The history and development of cancer treatment have opened an array of possible avenues.

## 3. Current Approaches to Cancer Treatment

### 3.1. Implementation of Hirudin in Cancer Treatment

**Figure 3:**
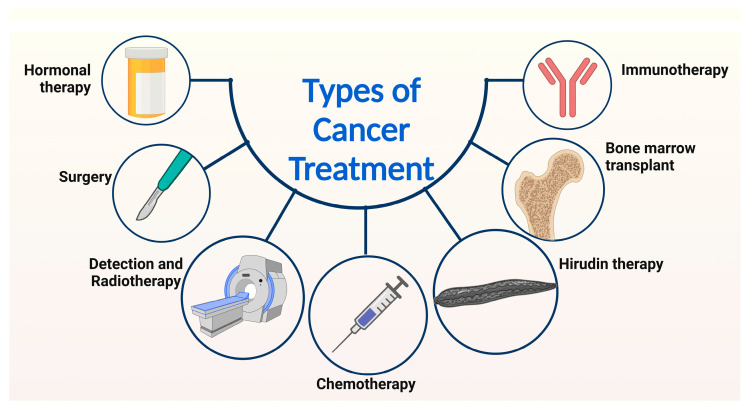
Presents an overview of the diverse methods employed in cancer treatment. A range of approaches, including bone marrow transplant, hormonal therapy, surgical intervention, immunotherapy, hirudin therapy, chemotherapy, and radiotherapy, are utilized to address cancer. Notably, this review focuses on the discussion of the current strategies for cancer treatment, specifically hirudin therapy, chemotherapy, and radiotherapy.

Hirudin originates from similarly related homologous peptides extracted from cranial salivary glands of medicinal leeches and is widely recognized as a thrombin inhibitor [58]. However, recent studies have indicated that hirudin has other capabilities, such as anti-tumor effects, anti-fibrosis properties, and wound repair [59] (Figure 3). Further studies indicated that hirudin can facilitate anti-tumor effects in glioma tissue, hepatocellular carcinoma tissue, hemangiomas, and nasopharyngeal carcinoma tissue. Hirudin’s anti-tumor effects include suppression of invasion, proliferation, migration, and metastasis of tumor cells. Additionally, hirudin stimulates apoptosis and hinders tumor growth by downregulating the following signaling pathways: HGF/C-met; VEGF/VEGF-R; and ERK. Hirudin interferes with the VEGF/VEGF-R angiogenesis signaling pathway by downregulating VEGF mRNA expression. Hirudin has the potential to decrease the angiogenesis-related expression. Tumorigenic growth is promoted by the increased expression of hepatocyte growth factor (HGF) and the mesenchymal–epithelial transition factor (c-Met). The overexpression of these factors upregulates the HGF/C-Met pathway. Upon treatment with hirudin, C-met expression significantly decreased, and inhibitory effects on mice tumor cells were exhibited.

Hirudin is also often used as a supplemental therapy in cancer patients receiving radiation therapy. This is because many patients face a significant risk of thrombosis while receiving conventional cancer treatments. Recognizing this, one group of researchers attempted a targeted hirudin delivery to mitigate thrombotic complications in cancer treatments by synthesizing and applying platelet-covered nanocarriers [60]. MnOx/Ag2S nanoflowers were used as the main structural framework for the nanoparticulate system. The surface of the nanoflowers was modified with platelet membranes to prolong blood circulation and ensure an accurate thrombus targeting [60]. The synthesized nanoparticulate system was able to sustain the release of hirudin and eliminate thrombosis in conjunction with anti-tumor therapy. Extensive studies indicated that the nanoparticulate system effectively delivered hirudin to thrombus-prone sites and released hirudin under near-infrared light radiation. When this occurred, thrombus sites were removed. Further studies indicated that this system could prohibit the progression of tumors and increase the life expectancy of mice with thromboembolic complications. Additionally, the MnOx integration into the structural framework was able to react with glutathione (GSH), abundantly found in tumor microenvironments, and release Mn^2+^ in tumor cells. The released Mn^2+^ then reacted with the prevalent concentrations of H_2_O_2_ in the tumor microenvironment to generate hydroxyl radicals through Fenton-like reactions. The hydroxyl radical then exhibited damage to tumor cells and induced cell apoptosis. Overall, the nanoplatform was able to exhibit synergistic functions by inhibiting thrombin activity and promoting antitumor therapy by depleting GSH to form toxic hydroxyl radicals to stimulate tumor cell apoptosis. Therefore, the in vivo results showed that tumorigenic mice treated with the synthesized nanoparticulate systems experienced prolonged survival times.

One limitation faced with hirudin treatment is its short half-life in the bloodstream. This property compromises the therapeutic efficacy of hirudin. To overcome this, research groups have engineered hirudin–bovine serum albumin (BSA) NPs characterized by desolvation techniques [61]. The hirudin-BSA NPs had improved sustained, controlled release of hirudin. These results indicate that the hirudin-BSA NPs have the potential to be applied to biomedical clinical therapies. Another research study manufactured polydopamine-fitted TiO_2_ nanotubes to prolong hirudin release and enhance hemocompatibility in vitro and in vivo. The aforementioned studies highlight the amplified therapeutic potential of synergizing conventional hirudin treatment with nanomaterials. Thus, the integration of nanomaterials for applications in cancer treatment offers a promising approach to improving conventional therapies.

### 3.2. Progresses in Detection and Therapy

In recent years, advancements in cancer diagnostics have allowed for earlier detection and improved prognosis factors for cancer patients [62]. Key advancements in cancer detection include screening tests, biomarkers, imaging technology, liquid biopsies, and the employment of artificial intelligence. The widespread use of screening tests, such as colonoscopies and mammograms, has allowed for the diagnosis of cancer before the presence of symptoms. Biomarkers are molecules that can indicate the presence of cancer in the body. Through extensive studies, research groups have identified various biomarkers for different types of cancer. For example, abundant concentrations of prostate-specific antigens can often be linked to prostate cancer, while the presence of human papillomavirus indicates cervical cancer. Screening methods such as blood tests enable the detection of biomarkers. Advances in imaging technology, such as magnetic resonance imaging (MRI), computed tomography (CT), and positron emission tomography (PET) scans, have improved the ability to detect and diagnose cancer (Figure 3). These imaging techniques can provide detailed images of the body, which aid in the ability to identify tumors and other cancer markers. Liquid biopsies are non-invasive tests that analyze samples of blood to detect the presence of cancer cells or genetic mutations associated with cancer. Liquid biopsies can detect the presence of cancer cells, even when the tumor is not visible on a traditional imaging test. More recently, artificial intelligence and machine learning have been used to analyze images from various sources, including radiology, pathology, and genomics. Research studies have confirmed that artificial intelligence can help detect cancer with high accuracy, as well as predict treatment outcomes and patient prognosis.

Radiation technology can be utilized in biomedical diagnostic and therapeutic applications. Radiation has extensive applications in biomedicine for imaging and therapy purposes. Specifically, ionizing radiation is useful for radiological imaging, cancer radiation therapy, and radionuclide imaging [63]. Radiation transmitting optical wavelengths is particularly applicable for localized imaging as well as photodynamic therapy, while longer wavelength radiation has effective Magnetic Resonance Imaging (MRI) capabilities. Radioluminescence conjugates optical and ionizing radiation and is utilized for radionuclide imaging, monitoring radiation therapy, stimulating phototherapy, and investigating the efficacy of nanoparticle-based diagnostics and therapeutics [63].

An emerging application of radiation is utilizing Cerenkov radiation from radionuclides and radiotherapy for optical imaging purposes [63]. Conjugating Cerenkov luminescence imaging (CLI) with radiopharmaceuticals, such as F-fluorodeoxyglucose, holds great potential to supplement sentinel node biopsies, monitoring tumor growths, and tumor imaging [64,65,66,67]. CLI is comparable in function to Positron Emission Tomography (PET) scans [63]. Additionally, CLI is an effective radiotracer for breast cancer and gastrointestinal tract (GI) tumors. Cancer phototherapy integrates photosensitizers for cancer phototherapy [64,65]. Photosensitizers are drugs that are activated when presented with a light stimulus [64]. Under optimal conditions, photosensitizers accumulate inside cancer tissue and aid in the generation of reactive oxygen species to stimulate oxidative damage and apoptosis in carcinogenic cells [68]. Using synergized titanium-oxide NPs with photosensitizers, a revolutionary solution for cancer therapy can be developed based on the current research [63,69]. Another radiation-based treatment involves scintillation, which is luminescence stimulated by ionizing radiation and has various biomedical applications [70]. Bulk inorganic scintillators are necessary for surgical gamma probes, nuclear imaging detectors, as well as X-ray imaging [71,72]. Organic scintillators are utilized in surgical probes and aid in liquid scintillation devices and radiotherapy dosimeters [73]. Additionally, they have applications in imaging the in vivo dosimetry and monitoring biological mechanisms. Another application of radiation is radioluminescence microscopy, which is an imaging device for beta-emitting radionuclides on a cellular level, which is not possible with PET imaging [63]. Radioluminescence microscopy is utilized to study metabolism, transgene expression, and cell proliferation in human carcinomas [74,75].

### 3.3. Chemotherapy

The overall goal of chemotherapy is to hinder cell proliferation and tumor cell mitosis [62]. Intervention with these cellular processes will thereby reduce invasion and metastasis. Conventional chemotherapeutics affect the synthesis of biologically relevant macromolecules and the function of DNA, RNA, and protein synthesis in tumorigenic cells (Figure 3). Current chemotherapeutics commonly target the S phase or M phase of the cell cycle and pose inhibitory effects (Table 1). For example, vinca alkaloids and taxanes function by preventing mitotic spindle formation and interfering with the M phase of the cell cycle. Combination chemotherapy is commonly prescribed to cancer patients with the purpose of targeting different genes, receptors, and signal transduction pathways. These combination therapies include different classes of drugs that inhibit growth, impede cell signaling, interfere with angiogenesis pathways, and suppress checkpoint protein degradation. Combination chemotherapeutic drugs are adjusted based on the evaluation of the following principles. The fraction kill hypothesis ensures that drug doses are formulated to kill a designated fraction of tumor cells, regardless of tumor size. The Goldie–Coldman hypothesis considers that cancer cells can acquire spontaneous mutations that result in drug resistance. While most cancer drugs described above interfere with metabolic processes and aim to restore cell cycle checkpoint mechanics, drugs such as cisplatin and oxaliplatin, pose therapeutic effects by functioning as cytotoxic metal compounds. Cisplatin, otherwise known as cis-diammine dichloroplatinum (II), is composed of a platinum (II) complex conjugated with chlorine ligands in a cis configuration. Due to its metal-based structure, cisplatin is widely recognized as a highly cytotoxic cancer medication. Thus, cisplatin administration doses must be closely controlled to avoid damage to biological tissue. Cisplatin exerts anti-tumor effects by forming cross-links with DNA that stimulate cell cycle arrest at S, G1, G2-M, and apoptosis [62].

### 3.4. Surgery

In the past, surgical intervention stood as the sole recourse for all types of cancer. While advancements in medical technology and procedures have bolstered the efficacy of oncological surgeries, the absence of optimal tumor imaging techniques hampers the effectiveness of surgery as the primary cancer treatment [76,77]. The less-than-ideal outcomes arise from the surgeons’ inability to completely excise the cancerous tissue due to inadequate visualization. Further, surgery and the concurrent inflammatory response have been recognized as concerns in cancers, taking advantage of the patient’s compromised state to metastasize soon after the operation [78]. Considering these challenges, intraoperative fluorescent imaging has emerged as a solution to facilitate improved visualization of the cancer site and enable precise and comprehensive removal of all malignant tissue [77]. Despite these inherent challenges, surgery has come a long way and continues to advance as a trusted and executed element of modern medical practices and continues to be a common approach to cancer remediation [78].

### 3.5. Hormonal Therapy

Hormonal therapy, alternatively known as endocrine therapy, is employed in the treatment of cancers that rely on hormones for their growth [79]. Breast and prostate cancers are most commonly seen as responsive to such treatments [80]. Some notable hormones involved in these particular cancer systems include progesterone, estrogen, testosterone, oestradiol, and androgens. These endocrine therapies often serve as complementary or primary treatments, effectively inhibiting or stalling cancer cell proliferation and mitigating symptoms in cases where alternate treatments like surgery or radiation are not viable. This therapeutic approach can precede a major treatment to reduce tumor size (neoadjuvant) or follow it to minimize the risk of cancer recurrence or spread (adjuvant), alongside its inherent ability to combat cancer cells influenced by hormones [80]. This therapy has been deemed efficient in both primary and metastatic conditions [80]. The two distinct forms of hormone therapy function either by using specific hormones to obstruct the body’s hormone production or by interfering with hormone activity within the body.

Hormone therapy can be administered through various means, including orally or via intramuscular injection. In some cases, the removal of the organ responsible for hormone production is considered a viable option as it deprives the system of the problem hormones [79,80]. For females, this typically involves an oophorectomy (ovary removal), while for males, an orchiectomy (testicle removal) is performed. There are different treatment approaches when it comes to pre- and post-menopausal cases, with pre-menopausal cases favoring the aforementioned castration and post-menopausal cases taking a more supplemental approach [80]. The impact of endocrine therapy tends to differ significantly from one patient to another. There is a high likelihood of patients developing resistance to the hormones, contributing to eventual readvancement of the cancer that once responded well to the treatment. This common occurrence is combatted by additional hormone introductions as well as chemotherapy [80]. Notwithstanding the varied efficacy and unpredictable consequences for each individual, certain common side effects manifest differently based on gender. Both men and women may experience symptoms such as hot flashes, fatigue, and nausea, among others (Institute 2015). This treatment varies in long-term effectiveness but provides a simple and non-toxic approach to hormone-related cancers [80].

### 3.6. Bone Marrow Transplant and Stem-Cell Therapy

Stem-cell therapy has demonstrated its effectiveness as a treatment option for various complications of cancer, both directly and indirectly. While these treatments are applicable in conjunction with others, such as chemotherapy and radiotherapy, stem cells are also frequently employed to alleviate the repercussions that cancer and its treatments have on the body [81]. Whether affected by the treatment itself or by the cancer, the body’s blood supply and immune system can experience regeneration with the aid of stem-cell therapy [82].

Cancer stem cells (CSCs) are thought to be derived from mutations in healthy stem cells. Renowned for initiating the onset of cancer and the disease’s resistance to therapy, CSCs have recently become a target of numerous anti-cancer treatments [83]. These strategies have been bolstered by the recent identification of CSC biomarkers, facilitating the advancement of these anti-cancer interventions. These undifferentiated cells contribute to the rapid proliferation, dissemination, and potential tumor development in cancers [84,85].

Found in the bone marrow, mesenchymal stem cells (MSCs) have been explored as a potential cancer combating tool. MSCs can differentiate into multiple specialized cell types and are known for their tissue regenerative abilities [84]. Their great potential is derived from their inherent ease of replication as well as differentiation into a multitude of cell types for immune regulation [86]. These multipotent stem cells can use paracrine systems to influence the growth and development of CSCs. These MSCs have been found to provide targeted action toward cancer cells, as well as the ability to transport interleukins, chemokines, and other natural immune activators to enhance the cancer-fighting abilities of the human immune system [87]. Despite the promising recognition of attenuation, some studies have seen exacerbated proliferation and metastasis of various cancers following the use of MSCs as a treatment [88,89,90,91]. Despite a multitude of studies, the varied results and wide range of research contribute to a deficit of understanding of MSC therapy as a whole [87,92].

In addition to non-cancer-related applications, Hematopoietic stem-cell (HSC) transplants are most commonly seen among treatments for leukemia, multiple myeloma, and lymphoma. Each of these cancers involves abnormal production of cells in the bone marrow. HSCs are known for their ability to differentiate into any blood cell type [84]. HSCs are typically applied in conjunction with other treatments such as chemotherapy, immunotherapy, or radiation. However, one study explored their use in mimicking innate immune cells, such as invariant natural killer T cells (iNKT), with promising results [93].

Neural stem cells (NSCs) are derived from the CNS and have also been recognized for their ability to regenerate themselves as well as new glial cells and neurons. They have also been studied as potential treatment elements of various cancers, with results speaking to the ability of these cells to hinder progression and metastasis [84,94,95,96]. Other studies also speak to the ability of these NSCs to be tailored to a specific tumor system for greater treatment specificity. These cells are expected to be useful not only in brain-related cancers but also show promise for the treatment of lung, breast, and prostate cancers [97,98].

Pluripotent stem cells (PSCs), previously and controversially derived solely from Embryonic stem cells (ESCs), show great promise for cancer treatment and regenerative medicine. The discovery was that previously differentiated cells could be returned to a state of pluripotency with the help of transcription factors, creating induced pluripotent stem cells (iPSCs) [99,100]. PSCs such as ESCs and iPSCs have the unique ability to mature into, with the exception of placenta, any cell type in the body [84]. Studies have revealed the ability of iPSCs to specify their differentiation to immune cells that are specific to a type of tumor (i.e., Natural killer (NK) and T cells) [101,102,103].

### 3.7. Immunotherapy

As of 2020, ongoing advancements were being made in the field of biomarker testing for cancer immunotherapy [104]. Given the varied response of cancer patients to immunotherapy, the role of these biomarkers is crucial in determining the suitability of such treatments for individual patients and the future of cancer immunotherapies [104]. Employing biomarkers from the patient’s blood and tissue remains the optimal approach for devising an immunotherapy treatment plan for specific cancer types [105].

Cancer immunotherapy operates based on an understanding of the adaptive immune system, focusing on the cancer immunity cycle. This cycle commences with the release of immunogenic neoantigen proteins by apoptotic and necrotic cells within the tumor region. Subsequently, these cells are recognized and engulfed by dendritic cells, which then migrate to the lymph nodes to activate tumor-specific T cells [105]. While immunotherapy can be highly effective, maintaining the integrity and continuity of the immunity cycle is crucial to prevent potential tumor growth and improper immune responses. Thus, comprehending the complexities of the cancer immunity cycle and strategically applying immunotherapy within the immune sequence is of paramount importance. The adaptive immune system, involving the rearrangement of B and T lymphocytes to generate a targeted response to antigens, is utilized to stimulate the body’s response to a tumor [105]. In simpler terms, immunotherapy assists the body in inducing the regression of cancer by introducing new antigens into the system and reinforcing immune reactions against foreign or malignant invaders.

Immunotherapy encompasses various treatment modalities. One approach involves the use of specific antibodies to unlock the full immune potential of T cells, effectively removing the brakes on their anti-tumor activity. For instance, antibodies are utilized to block immune checkpoints like cytotoxic T-lymphocyte antigen 4 (CTLA-4) [106]. Another approach entails extracting the patient’s T cells, genetically modifying them to enhance their efficacy against tumors, and then reintroducing them into the patient’s system [107]. Currently, two drugs, pembrolizumab and nivolumab, have received FDA approval for cancer immunotherapy. Initial studies indicated that cancers characterized by a high density of tumor mutation exhibited the most favorable response to immunotherapy treatments, although this correlation was not consistently observed [105]. Furthermore, in patients with melanoma and non-small cell lung cancers, immune checkpoint blockade therapy demonstrated increased effectiveness as the neoantigen burden heightened. These findings have led to further research suggesting the use of specific neoantigens in cancer vaccines and more precise biomarkers for predicting the effectiveness of cancer immunotherapy [105].

## 4. Radiotherapy

**Figure 4:**
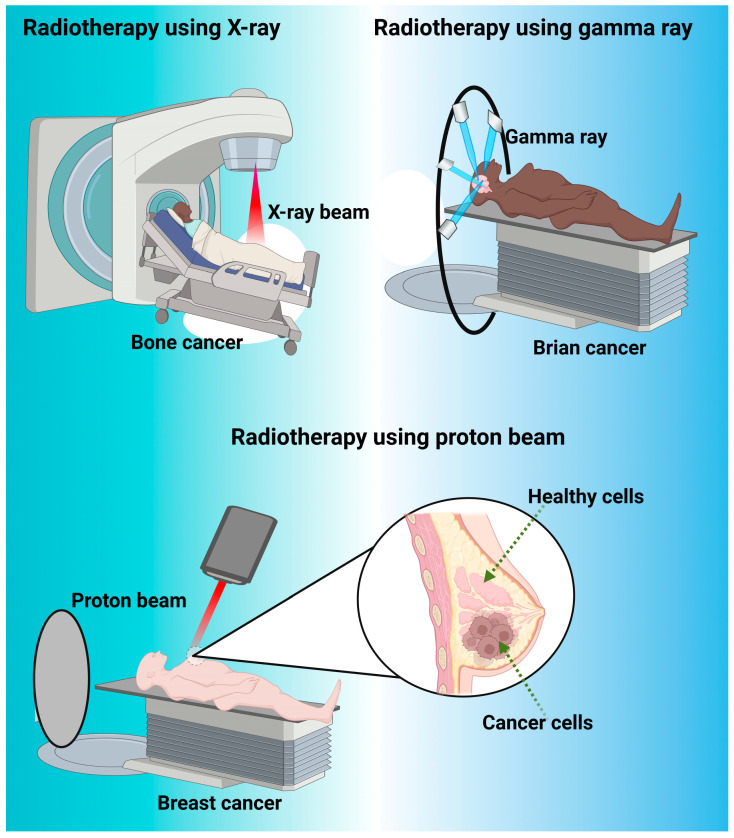
Represents the different type of radiotherapy such as X-ray beam, gamma ray and proton beam used for cancer treatment. X-ray beams are com-monly used for cancer treatment in a medical procedure known as radiation therapy or radiotherapy. In radiation therapy, high-energy X-ray beams are directed at the cancerous cells to damage their DNA and inhibit their ability to grow and divide. This helps to shrink tumors or eliminate cancer cells altogether. Gamma rays are also used in cancer treatment as a form of radiation therapy. Gamma rays are high-energy elec-tromagnetic waves that are produced by radioactive sources or specialized machines called linear accelerators. They are similar to X-rays in terms of their ability to damage the DNA of cancer cells, thereby preventing their growth and division. It is often used for treating certain types of tumors, particularly those located in the brain and nervous system. It’s a precise and non-invasive method that focuses a concentrated dose of gamma rays on the tumor while minimizing damage to surrounding healthy tissue. Proton beam is also another type of radiotherapy, which is a specialized form of radiation therapy used for cancer treatment. Proton beams are high-energy particles that can target cancer cells with precision while minimizing damage to surrounding healthy tissues. This makes proton therapy particularly useful for treating certain types of cancer and tumors located in sensitive areas, such as critical area like spinal cord, brain, eyes, and pediatric cancer patients.

Radiotherapy involves the use of high-energy particles or waves to damage or kill cells that are potentially dangerous to the body (Figure 4). Radiotherapy is a core part of the current cancer treatment and can be used in the future for applications such as pain control (Table 2) [108]. Radiation therapy commonly presents itself in two forms: external beam radiation therapy, in which the radiation required for the therapy comes from an external source that aims the radiation at the target location; and internal radiation therapy (also known as brachytherapy), in which the radiation source is placed in your body near the target location [109].

### 4.1. Radiotherapy Success

Radiotherapy has dominated as a cancer treatment for many years. Unlike other traditional cancer medicines, radiotherapy provides a more local therapeutic as opposed to exposing the whole body to a chemical that may occur from ingestion or negative effects from invasive surgery [110] (Figure 4). An experiment performed on the efficiency of radiotherapy in cancer treatment found that when compared to surgical treatment, a hybrid system of surgical and radiation therapy lowered the relapse rate from 54% to 24%, as well as extending the average life length of patients [116]. This study demonstrated the potential benefits of radiotherapy over normal therapeutic practices and showed the potential for hybrid treatments to help achieve better medical outcomes. An additional study analyzed pituitary adenomas in response to three treatments: medical therapy; radiation therapy; and surgical therapy. The results showed that radiation therapy resulted in nearly 50% of patients remaining free of reemergent adenomas after 10 years. Additionally, radiation therapy allowed for the treatment of adenomas that were refractory or otherwise not amenable to surgery [117]. Radiation therapy not only removes pituitary adenomas in the short term but also provides a baseline for preventing their re-emergence in the future [117].

### 4.2. Modern Radiotherapy

In modern radiotherapy, there are many different techniques to enhance or change the goals of a radiotherapy treatment (Figure 4). One example of this is pulsed/reduced rate dosing. This treatment is administered following the progression of the cancer significantly up the body. One case study performed on this treatment showed that while initially treating the patient with surgery, 54 Gy was delivered in 1.8 Gy dosages for his Grade II astrocytoma, and following his progression to Grade IV astrocytoma, the dosage was reduced to 50 Gy with 2.0 Gy dosages [118]. The main reason for using different dosages following the progression of the illness is due to the reirradiation of the tumor bed and the surrounding tissue area. Continuously providing high amounts of radiation to the tissue bed has shown to be extremely negative for tissue necrosis and can result in the death of surrounding cells and tissue. Therefore, decreasing the amount of radiation that was supplied provided tangible results without causing necrosis to the same vast extent [119]. Additionally, a separate study has shown the use of hyper-radiosensitivity of tumor cells, as well as the reduced normal tissue toxicity in normal cells, to provide more positive clinical outcomes. This study looked specifically at recurrent cancers and found that using the lower dosage rates, the body can take advantage of the reirradiation of the tumor cells but also the increased regenerative speed in normal cells at a low radiation level. This allows the body to heal while also combating cancer with a safer alternative [120].

Another facet of modern radiotherapy is combinatorial therapy. This treatment involves the use of one or more methods working together in a therapeutic setting to provide more positive results for a patient, especially with a lack of response to a singular method. Whereas the combination of radiotherapy with treatments such as surgery or immunotherapy can see better results, regression of cancer is less likely with monotherapy [121]. In this study, the therapeutic plan was to combine radiation therapy with immunotherapy, both of which provide positive results when utilized separately, to combat nasopharyngeal carcinoma. Immunotherapy lacks the ability to work beyond the small subset of patients with existing T-cell responses, whereas radiation therapy lacks the ability to truly make the regression of cancer sufficient to keep it from progressing. However, when combined, this study cited the benefits of immunotherapy in preventing the relapse of cancer in patients and keeping them healthy for longer and reported more positive clinical outcomes as a whole [122]. Recent studies have examined the therapeutic potential of inorganic NPs targeted to modify the immune response to cancer cells. One recent study synthesized IR-68 lonidamine NPs functionalized with albumin to reactivate immunotherapy mechanisms via the programmed cell death ligand 1 (PD-L1) [123]. The synthesized nanoparticle was found to be effective in regulating PD-L1 expression and oxygen concentrations and facilitating photodynamic therapy. These properties stimulated T-cell mediated tumor cell apoptosis. Furthermore, in vivo studies conducted with the R-68 lonidamine albumin NPs to examine their metastatic tumor-targeting capabilities yielded positive results. Currently, researchers are focused on overcoming the following challenges regarding precise tumor-targeting therapies: enhancing the biocompatibility of therapeutics and addressing the immune system evasion properties of tumors [124]. Various research studies have noted positive results when examining the potential of inorganic NPs to stimulate the immune response against tumors. One study found that endocytosis of calcium NPs induced immunogenic-mediated mitochondrial deterioration and cell apoptosis [124,125]. Other studies also noted that manganese and zinc NPs were capable of inciting adaptive and innate antitumor immune mechanisms by stimulating the STING immunogenic pathway [124,126]. Effective anti-tumor immune responses were achieved when cancer cells were treated with carbon nanotubes, silicon NPs, black phosphorous nanosheets, copper–zinc NPs, gold–silver NPs, and zinc–carbonate hydroxyapatite nanocrystals. Furthermore, inorganic NPs can promote potent anti-tumor immunogenic responses when functionalized with biological membranes. Additionally, various immune checkpoints are modulated with different formulations of inorganic NPs. Comprehensive research studies have shown that aluminum oxide NPs can upregulate the expressions of CD80, CD86, MHC-1, and MHC-2 molecules in dendritic immune cells [127]. The overexpression of these checkpoint molecules stimulates the secretion of interferon-γ to inhibit tumorigenic growth. Other studies have shown that the synergistic therapy of black phosphorous quantum dot nanovesicles and nonionizing radiation facilitated the expression of immune excitatory molecules such as CD80, CD86, MHC-1, MHC-2, and dendritic cell activation. Widespread research studies have found that gold NPs (AuNPs) can incite anti-tumorigenic immune responses by upregulating IL-2 levels, TNF-α, and IFN-γ immune checkpoint modulators [128]. The upregulation of these checkpoint modulators further stimulated MHC-2 macrophage expression and CD4^+^-mediated immune response. Another current treatment is combinatorial drug treatment in cancer patients, combining drugs that are complementary in their targets. A study was conducted on the use of CDK-targeting drugs with growth factor-targeting drugs as a possible therapeutic technique against cancer. This study found that the use of multiple drugs lowered the likelihood of a cancer becoming resistant to the drugs. This allowed for the dosages to be increased at a much slower rate and for the drugs to affect the body less despite their high dosages. This study also found that using an immune-stimulating drug could actually stimulate the body to use its own T cells to fight off cancer, allowing for limited effects of the drugs in the body, as there were noticeably fewer foreign substances within the body [122].

### 4.3. Proton Therapy

Proton therapy is another notable development in cancer medicine. Proton therapy provides tangible benefits over normal forms of therapy due to its ability to provide a safer distribution of radiation through the tissue, thus facilitating a safer therapeutic process as a whole. This means that dosages can be increased past those of conventional radiotherapy and can potentially be used for reversing the progression of cancer and finding a more permanent cure (Figure 4). Proton therapy provides a higher curative dose with similar side effects to current radiotherapy treatments [129]. Radiotherapy is significantly more expensive, however. The average cost of a proton therapy facility is about EUR 62.5 million (USD 68 million), which corresponds to an increase in patient treatment prices of almost 93% [130]. Not including the additional travel costs for patients and lodging costs, the technology is vastly too expensive for the general public to use [131].

Nanoparticles have also been integrated with ionizing radiation for diagnostic imaging and therapeutic approaches. Nanoparticle therapy facilitates X-ray absorption in corresponding tissues [132]. Furthermore, physical radio-sensitization is utilized to facilitate nanoparticle luminescence for biomedical purposes (Table 3). Radio-luminescent NPs are synthesized with materials that possess the property of luminescence when induced with ionizing radiation. The conjugation of ligands (i.e., antibodies, peptides, small molecules) on the NPs’ surfaces allows for efficient imaging and therapeutic opportunities. Radio-luminescent probes, such as quantum dots, metal nanoclusters, metal–organic frameworks, and polymer dots, can be synergized with conventional nanoparticle therapies to improve the efficacy of diagnostic and therapeutic approaches [133,134,135,136]. X-ray-activated photodynamic therapy integrates scintillators with radiotherapy to transduce photodynamic therapy and increase the efficacy of radiotherapy by converting X-ray energy into a light stimulus for activation of photosensitizer [137,138,139,140]. By synergizing photosensitizers with radiotherapies, successful necrosis and suppression of osteosarcoma tumors in mice can be achieved [141]. Overall, these literature surveys and experiments show that radiation is clinically applicable for diagnosing and treating various pathogeneses.

## 5. Radio sensitization Using Organic Molecular Compounds

While traditional radiation therapy is quite productive against several types of cancers, it is still not as effective against certain cancers such as pancreatic, lung, and brain. These variants are more resilient to radiation in lower doses and often require higher dosages of radiation, which can be detrimental to the surrounding healthy tissues [151]. To combat the clinical limitations of radiation therapy, strategies are in development to enhance the tumor damage associated with radiation. Radiosensitizers (also known as radio enhancers) either work to directly damage DNA through the formation of free radicals or indirectly through dysregulation of cell cycle checkpoints in tumor cells. The latter utilizes conventional chemotherapy agents as the dysregulation compound in conjunction with radiation therapy [12]. Examples of this process include the utilization of alkylating agents (such as cisplatin), antimetabolites (such as gemcitabine and 5-fluorouracil), anti-tumor antibiotics (such as doxorubicin), and fluoropyrimidines. These drugs are often utilized independently of radiation, but recent trends in clinical applications have seen the advent of chemoradiotherapy, where traditional radiation treatment is combined with chemotherapy drugs [152].

## 6. Inorganic Nanomaterials

### 6.1. Gold Nanoparticles

**Figure 5:**
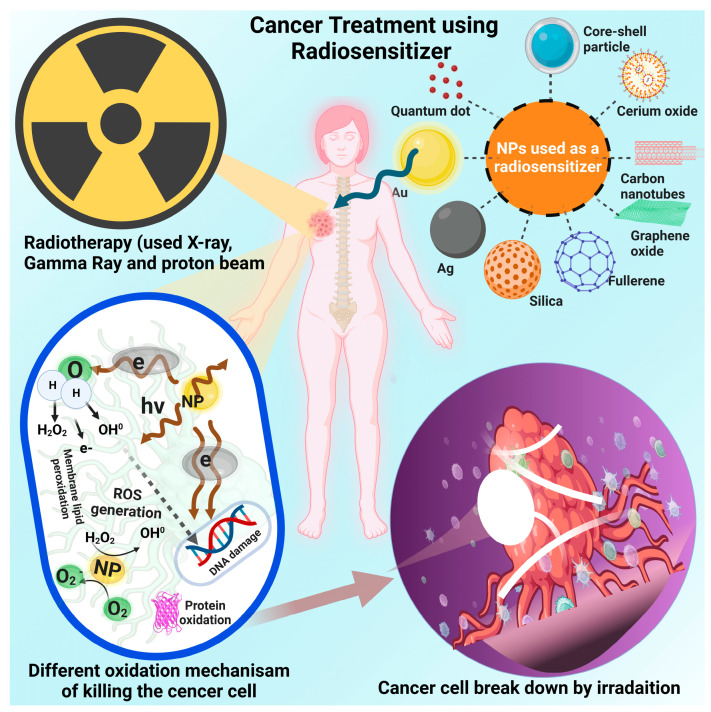
Represents the cancer treatment using radiosensitizer (inorganic NPs). For cancer treatment how inorganic NPs are used as radiosensitizers, (i) Engineered, high atomic numbers of NPs result in increased absorption of ionizing radiation such as X-rays, photon, or gamma rays. When these NPs are introduced into cancer cells, they amplify the absorption of radiation within the tumor, leading to enhanced local radiation dose and damage, (ii) the metal-based NPs have the ability to generate reactive oxygen species (ROS) when exposed to radiation. (iii) inorganic NPs can enhance the deposition of energy from radiation in close proximity to the cancer cells’ DNA. This leads to an increased occurrence of double strand breaks and other types of DNA damage that are difficult for cancer cells to repair, (iv) inorganic NPs can be function-alized with targeting ligands that direct them specifically to tumor cells or the tumor microenvironment. This targeting ensures that the NPs accumulate more in cancerous tissues than in healthy tissues, maximizing their radio sensitizing effect on the tumor, (v) inorganic NPs can be used in combination with other therapies, such as chemo-therapy or immunotherapy, to create synergistic effects. (vi) some inorganic NPs have imaging properties that allow them to be tracked in the body using imaging techniques like MRI or CT scans. This provides real-time information on nanoparticle distribution and treatment efficacy, (vii) there are also challenges to address, such as ensuring bio-compatibility, minimizing potential toxicities, and achieving consistent and reliable results while using of inorganic NPs as radiosensitizers. Ongoing research is focused on optimizing nanoparticle design, understanding their behavior within the body, and conducting preclinical and clinical studies to evaluate their safety and efficacy for cancer treatment.

Gold NPs possess highly unique characteristics that help them become versatile tools in the medical field [153]. These NPs are known for their ease of synthesis, exceptional biocompatibility, chemical stability, and the capacity to enhance local radiation doses. Recognizing these qualities marks a pivotal breakthrough in advancing cancer treatment (Figure 5). A prior study revealed that when cells uptake AuNPs, they can significantly enhance radio sensitization, leading to an increase in energy from 1.01 KeV to 2.11 KeV in vitro (Table 4) [154,155]. These findings have been corroborated by other studies, showing a substantial rise in apoptosis among cancer cells that absorbed AuNPs [156]. Moreover, research suggests that greater radiation enhancement is achievable at lower energies, expanding the practical applications of these NPs and opening new avenues in the biomedical field [156]. Another common aspect of gold nanoparticle research has been the assessment of potential toxicity in the body. In an in vivo study, reports emerged of hematological toxicity (blood), nephrotoxicity (kidney), hepatotoxicity (liver), immunogenicity, as well as oxidative and inflammatory responses resulting from AuNPs. Exposure to AuNPs can result in organelle damage, mutagenesis, apoptosis (programmed cell death), oxidative stress, DNA damage, and protein misregulation [157]. Gold is reportedly somewhat cytotoxic, with another in vitro study suggesting it caused nearly 23% cell death in epithelial and primary cells exposed to it [157].

Gold NPs show great promise for the medical field with their ability to be uptaken into cells, an uncommon trait for most NPs. Particularly, those with a diameter under 12 nm can penetrate the blood–brain barrier [158]. Investigations have revealed that the accumulation of AuNPs in the body is size-dependent, with particles under 50 nm in diameter easily traversing membranes and dispersing in and out of cells. Nanoparticles that penetrate cells can fully leverage their unique traits and capabilities. Gold NPs under 15 nm in diameter were found in an extremely wide array of locations, including the heart, brain, liver, lung, kidney, stomach, spleen, and bloodstream. This widespread distribution showcases their capacity to navigate the body effectively, setting them apart from many other biocompatible NPs, which are more limited in use. Other metallic NPs tend to become absorbed by the liver, which decreases their benefits in the upper body and affects their efficacy among neurological issues [159].

Gold NPs show promise in cancer treatment, particularly in radio-sensitization. A study using 10 nm colloids with functionalized PEG polymers demonstrated higher uptake in cancer cells than normal cells, suggesting the effectiveness of using AuNPs for targeted radiation therapy with reduced impact on healthy tissue [160]. Surface functionalization is increasingly common, enabling precise targeting of cancer cells while minimizing effects on the surrounding tissue [161]. Gold exhibits many promising attributes beyond its radioprotective potential. Its radiation-enhancing capabilities are already employed in cancer treatments, reducing the radiation dose required for cancer cells while sparing healthy cells. Gold’s unique ability for cellular uptake and systemic distribution sets it apart from most NPs, impacting the entire body rather than being limited to liver absorption, though it can have some toxicity concerns in human use [162,163].

Localized heating is another focus of research on AuNPs. Due to their easily tailored morphology and surface chemistry, AuNPs can absorb light energy to impose targeted photothermal damage upon cancer cells [162,163]. Most studies induce incident heating with lasers, mentioning the specific virtue of AuNPs in absorbing near-infrared light. Hastman and colleagues noted the advantage of nanoscale temperature therapy, as it can reduce systemic influence and focus treatment effects on the subcellular level [164]. Akouibaa et al. describe the useful localized surface plasmon resonance (LSPR) of AuNPs, which excite to produce a targeted heat source. This plasmon resonance is unique to gold on the nanoscale and is characterized by dipole oscillation of the valence electrons in the electromagnetic field of the corresponding energy source [165]. Moustaoui and colleagues investigated surface temperature increases in various gold nanoparticle shapes to determine their sizes and shapes for photothermal therapies [166]. They compared absorption values of nanospheres and nanourchins with theoretical calculations, finding that branched urchin-shaped NPs exhibited the highest photothermal damage capacity due to increased absorption and emission at branchpoints. Additional studies explored how these particles induce hyperthermia in cells by releasing ROS to trigger cancer apoptosis [166].

Phototherapy has recently been researched for efficacy in synergy with existing cancer treatments [166]. Mirrahimi et al. studied a local synergy of existing treatments using the phototherapy of AuNPs in radiotherapy and chemotherapy [166]. This study used AuNPs loaded with cisplatin in an alginate-based hydrogel. The combination of these therapies resulted in enhanced local heating and reduced tumor growth rates compared to radiation alone. In in vivo studies, mice injected with the gold nanoparticle cisplatin combination before laser irradiation experienced quicker heating and higher tumor temperatures. Gold NPs were essential for the therapy’s effectiveness in suppressing tumor growth in mice, with thermo-chemo-radio therapy leading to the highest subject survival rate. Other studies verified the efficacy of nano-photothermal immunogenic cell death in synergy with other cancer drug therapies such as cyclophosphamide, anthracyclines, and oxaliplatin [167]. One study discusses thermoplasmonics and the various methods by which gold nanoparticle absorption can be measured. In addition to classic infrared thermography, they delve into the use of thermosensitive liquid crystals and hydrogels for meaningful nanoparticle photo absorption data [166]. The electron transitions follow a light pulse to allow for the photon absorption of the nanoparticle, which emits heat by coupling with the electromagnetic wave due to its specific surface plasmon resonance. The effects of this process are dependent on the environment of the particle [166]. As studied, the tumor environment is receptive to apoptosis by photothermal therapy. The nano-aspect of photothermal therapy has been found to not only induce anti-cancer activity but also to exhibit the onset of enhanced immune system activity [167]. In comparing different gold nanosphere shapes, nanoshells/nanocages, nanostars, and nanorods, one study highlighted their distinct properties. The nanosphere has its absorption peak at 530 nm, but aggregation can shift this toward near-infrared for greater penetration.

Nanoshells and cages feature a gold layer around a dielectric core, often PEG-coated, commonly used in the near-infrared range. Gold nanostars exhibit plasmon hybridization at their core and easily tunable tips, enabling deep near-infrared absorption and tumor tissue accumulation. Silica surface layering of gold nanostars is recommended on account of the shape denaturation in heating. Gold nanorods, in particular, have gained recognition for their exhibition of two peaks in their surface plasmon resonance, generating peaks at 530 nm and in the NIR range [167]. This property inspired research on gold nanorod potential for simultaneous optical imaging and photothermal properties in a cancer system [167,168]. Multiple studies have attempted to enhance the photothermal properties of gold nanorods with various metals, including Pd and Pt [169,170,171]. Another recent study found gold nanoribbons made via seed-mediated synthesis and exposed to NIR outperformed nanorods in inducing MCF-7 cancer cell death [172]. In addition to the prolonged therapy release and specific cancer tissue-targeting ability of these NPs, each of the morphological variations in AuNPs shows promise for systemic immunotherapy [167,169,173].

More studies observed the efficacy of AuNPs in producing excess reactive oxygen species (ROS) as a means of stimulating cancer cell apoptosis. A recent study observed the character of peanut-shaped AuNPs and found them to disrupt the redox balance effectively, leading to ovarian cancer cell death. They found the gold nano peanuts to reduce the free thiol ROS scavengers and, thus, induce oxidative stress. Further investigations proposed that cell death results from the autophagy and apoptosis mechanism of the JNK signaling pathway, which is triggered by this stress [174]. This glutathione consumption and related ROS-induced apoptosis are further confirmed in another study that advocated for drug-free cancer nanotherapies [175]. One study employed IR-780 gold nanocages with a Pluronic F127 linkage. The Pluronic-linked nanocages exhibited a 16% increase in loading efficiency as opposed to the control nanocages and showed photothermal and apoptotic effects of up to 89.6 °C in the tumor environment, exhibiting a 1500 mm^3^ volume ablation capability [176]. Another study employed chitosan-coated AuNPs, finding their particles to selectively induce cell death via ROS production in a number of leukemic cancers [177]. Further research indicates the biocompatibility of gold nanoclusters with enhanced endocytosis, as well as their NIR efficacy in imaging, biolabeling, tracking, and cytotoxicity for diagnosis and treatment of chronic myeloid leukemia [178].

**Table 4:** Summary of studies discussing the utilization of AuNPs to enhance the radiosensitization effect of conventional radiotherapy for improved cancer outcomes.

Nanoparticle and Functionalization	Synthesis Method and Size	Experimental System	Radiation Utilized and Dose	Mechanism	Notable Results	Ref.
Spherical AuNPs in the center of a water cube	Simulated model, 10 nm to 150 nm in diameter	Tumor cell in the head in a spherical shape with a radius of 0.8 cm	X-ray radiation, 20 keV and 50 keV	Simulated tumor cells absorbed high amounts of energy in the presence of gold. During irradiation, gold increased the production of secondary electrons as well as photons in cancerous cells.	The absorbed energy for the 20 keV was higher than any other dosage. Irradiation in the presence of AuNPs was significantly better than irradiation alone.	[155,156]
AuNPs	Spherical, 0.18 nm	Tumor Cells	X-rays, 81 keV	There is a significant dose enhancement near the Au/Tissue interface due to the enhancement at a low Z/high Z interface.	Results showed an increase of about 550% in dose enhancement for the AuNPs.	[157]
Transferrin peptide targeted AuNPs	Modified Brust-Schifrin, 8.2 nm on average diameter	Human glioma cancer lines	Photosensitizers	Utilizing Tf_pep_ improves AuNPs specificity. PDT-killing efficacy is effectively reached.	Experimental system shows a successful delivery system to brain tumors utilizing AuNPs.	[161]
AuNPs	55 nm on average	dsDNA	Femtosecond laser excitation, 750 nm	The AuNPs can modulate the local rate of denaturation by forming a structure with single-stranded DNA.	The results showed that modulation increased nearly threefold during the use of gold nanoparticle intermediates.	[165]
2-d gold nanoribbons	Pre-synthesized by mammalian cells, 20 nm	MCF7 Breast Cancer cells	NIR irradiation, 0.6 W	The nanoribbons aided in many different areas, including SERS imaging as well as NIR hypothermia and potential for a new nanomedicine.	Nanoribbons using the seed-mediated method could potentially produce a state-of-the-art cancer therapeutic treatment.	[173]
Chitosan-coated AuNPs	Chemical methods, 3.7 nm on average	Chronic myeloid leukemia cell line (K562)	Not stated	Cell death was dependent on ROS production.	TheNPs induced selective cell death in leukemia cells.	[178]

### 6.2. Silver Nanoparticles

One unique characteristic of silver nanoparticles (AgNPs) is their local plasmon resonance frequencies that have a myriad of applications in nanocrystal development. One study integrated plasmon resonance to modulate nanocrystal tuning of nanocrystals in any visible or near-infrared region (Table 5). This approach enabled researchers to synthesize silver triangles with defined edges to function with enhanced efficacy in propagating a specific wavelength. Another study was conducted, attempting to combine monodispersed NPs with metal NPs. They found that the monodispersed NPs enhanced the sharpness of the local field. This has potential optical applications to generate thin films. These fabrications also have capabilities in non-linear fields, as they can tune the optical nonlinearity of nanoparticle films. Additionally, these NPs have optimal surface bioconjugation, which has various biotechnological applications. Silver NPs are toxic to the vast majority of viruses and bacteria while non-lethal to normal cells in small dosages (Figure 5). This property allows for AgNPs to be an ideal sterilization tool, especially from a medical standpoint. AgNPs have relevant biomedical applications in cancer therapy due to their ability to facilitate joule heating [179]. Joule heating is the process in which electrical energy is converted to heat energy to induce a state of hyperthermia in affected biomolecules. This effect works in conjunction with radiotherapy to amplify cancer cell apoptosis through the generation of ROS.

The utilization of AgNPs as radiosensitizers has gained widespread attention in recent years. One study synthesized AgNPs functionalized with Polyethylene Glycol (PEG) and Aptamer As1411 to amplify the radiosensitivity of glioma tumor cells, induce apoptosis selection of glioma cells, and mitigate the irradiation therapy side effects [181]. Extensive studies showed that the PEG and As1411 surface modifications significantly enhanced Ag nanoparticle uptake in glioma tumor cells. Additionally, the PEG-As1411-AgNPs successfully penetrated the core of tumor spheroids and facilitated amplified apoptosis rates. Overall, the synergistic therapy of AgNPs and X-ray irradiation prolonged the survival time of C6 glioma-bearing mice. In another study, researchers synthesized PEGylated graphene quantum dot-decorated silver nanoprisms by exploiting the non-covalent electrostatic interactions created between the AgNPs and the PEG-graphene quantum dots (PEG-GQDs) [182]. The goal of this study was to improve the efficacy of conventional radiotherapy by synthesizing a nanoparticulate system for applications in radio-sensitization for the treatment of colorectal cancer. X-ray irradiation at a dose of 2–10 Gy was tested on the HCT 116 and HT29 (relatively radiation-resistant) human colorectal cancer cell lines. PEG-GODs were attached to silver nanoprisms to enhance targeting and aid NPs uptake into colorectal cancer cells, making the HT29 cell line more receptive to conventional X-ray therapy. Detailed studies indicated that the conjunction therapy of conventional radiation and the administration of the synthesized NPs stunted colorectal tumor growth and prolonged the survival time when compared to groups treated with only radiotherapy. Another study synthesized AgNPs functionalized with anti-EGFR antibodies via the thermal reduction method to amplify the apoptotic effects of radiotherapy with the co-administration of the Ag-anti-EGFR NPs for applications in nasopharyngeal carcinoma. Experiments were conducted on human nasopharyngeal cancer cells with X-ray doses ranging from 0 to 8 Gy. Overexpressed EGFR in these cells enhances the uptake of synthesized NPs through receptor-mediated endocytosis. Results showed that the Ag-anti EGFR NPs inhibited nasopharyngeal carcinoma epithelial cell proliferation and stimulated their apoptosis via G2 cell cycle arrest [183]. One study fabricated cisplatin-loaded Ag NPs to assess the cancer therapeutic effects of Ag NPs in ovarian cancer cell lines [184]. Cisplatin was conjugated with AgNPs to improve its targeting and accumulation in ovarian cancer tissue. The AgNPs inflicted cytotoxic effects through DNA damage and ROS amplification on the A2780 and SKOV3 ovarian cancer cell lines, while the OVCAR3 cell line showed lower responsiveness to the AgNPs. Overall, the combination therapy of AgNPs and cisplatin displayed synergistic effects and resulted in a favorable cisplatin dose reduction in the treatment of ovarian cancer cells. Another study investigated the therapeutic and radio-sensitizing effects of AgNPs under 6 MeV X-ray radiation (dose rate: 200 cGy/min) [185]. AgNPs facilitated therapeutic effects in glioma cells by stimulating apoptosis and facilitating destructive autophagy. The combination therapy of AgNPs coupled with radiotherapy elicited significant anti-glioma effects in hypoxic U251 and C6 glioma cell lines. In another study, resveratrol-loaded AgNPs were conjugated with graphene quantum dots [186]. This study aimed to evaluate the therapeutic potential of combining AgGQDs with resveratrol for treating colorectal cancer. Experiments were performed on HCT-116 colorectal cancer cells exposed to 2 Gy of X-ray irradiation. AgGODS were surface-conjugated to enhance targeting and facilitate resveratrol delivery to colorectal cancer cells via receptor-mediated endocytosis. Results showed that the colorectal cancer cell viability was significantly hindered, and apoptosis was amplified when treated with X-ray irradiation and silver quantum dot-Resveratrol NPs. Overall, the synergistic treatment upregulated caspase-3 mRNA and decreased COX-2 protein expression in colorectal cancer cells. One study synthesized Ag NPs to evaluate the cytotoxicity of Ag NPs on the MCF-7 and MCT breast cancer cell lines under 6 Gy of gamma-ray irradiation (dose rate: 0.675 Gy/s) [187]. The Ag NPs were engineered using a novel rapid extracellular biosynthesis derived from the utilization of the fungus Penicillium aurantiogresium. Extensive studies showed that AgNPs displayed dose-dependent cytotoxicity and confirmed their ability to function as a potent radiosensitizer for gamma irradiation. Additionally, administration of silver NPs altered cell morphology, inhibited cell proliferation, and activated lactate dehydrogenase and caspase-3. Another study fabricated silver NPs synthesized with normoxic polyacrylamide gelatin and tetrakis hydroxy methyl phosphonium chloride polymer gel [188]. The objective of this study was to create an advanced dosimetric and theranostic nanoparticulate system to amplify dose distribution in cancer tissue and serve as an effective contrast agent in MRIs under 6–25 gamma-ray irradiation. At an ideal dose, the AgNPs facilitated polymerization of the polymer gel, resulting in an 11.82% increase in the accumulation of anti-cancer drugs in tumor tissue when 2 mL of AgNPs was used. Overall, the integration of silver NPs in the system increased the optical density of the drug delivery fabrication. In one study, AgNPs were functionalized with carbon nanodots and PEG. The study aimed to assess the potential of PEG-coated Ag-carbon nanodot NPs as a radiosensitizer for enhanced prostate cancer treatment with X-ray irradiation. PEG improved NPs affinity for prostate cancer cell accumulation, while carbon nanodots in the Ag nanostructure enhanced stability and radiosensitization. The combination therapy of X-ray irradiation and Ag NPs provided a synergistic effect and reduced prostate cancer cell survival by 50% [189]. One study synthesized bimetallic Au/AgNPs to assess the efficacy of the bimetallic NP system in functioning as a radiosensitizer during the radioembolization of liver tumors [190]. Experiments were conducted using N1-S1 hepatocellular carcinoma cells under external beam radiation (0–10 Gy) and 90Y TheraSphere exposure. The branched bimetallic Au/AgNPs synthesized from Bile acid, optimizing their surface area and catalytic activity. The bimetallic Au/AgNPs acted as a radiosensitizer by generating ROS, achieved through Au/Ag mediated electron transfer to intracellular oxygen. Results revealed that exposure to external beam radiation or 90Y TheraSphere, along with the bimetallic Au/AgNPs administration, significantly increased ROS production. This ROS generation induced oxidative stress and promoted apoptosis in hepatocellular carcinoma cells.

### 6.3. Silica Nanoparticles

Silica NPs (SiNPs) are spherically shaped nanoparticles that exhibit many unique surface properties that allow them to be easily altered for tailored use. Silica NPs have shown promise in the nanomedicine field due in part to their functioning surface chemistry, simple synthesis, protected circulation, stability compared to other nanoparticles, and low cost of production (Table 6).

Silica NPs have demonstrated significant radiosensitivity properties (Figure 5). Due to the relatively small size at which SiNPs can be functionalized and produced, they possess the capabilities of entering a cancerous cell and enabling cell signaling to help increase the effects of radiation treatment on the cells while limiting those effects on non-cancerous cells [191]. A study performed using amorphous SiNPs with hyperbranched poly(amidoamine) grafted onto the surface found that SiNPs could be used to increase the damaging effects of radiation on cancerous cells [192]. Results showed that the breast cancer cells, when exposed to radiation, were shown in significantly reduced presence [193]. A separate study found that when using mesoporous SiNPs, the cells could be functionalized with VPA and treated to recognize the overstimulation of folic acid in cancerous cells. This allows for an increased effectiveness of treatment at a lower radiation level, saving the non-cancerous cells from the serious side effects of radiation treatment. This method also increased cancerous cell targeting and allowed SiNPs to secrete VPA to help specifically target the cancerous cell line. Using uncapped and aminosilanized SiNPs, another study was able to increase the ROS in cancerous cells by 180% by limiting the effect in nearby cells to only 120% [193]. The synthesized SiNPs were used in puncturing through the cancerous cell’s mitochondria, allowing for a state of oxidative stress to take over in the cell. This study showed how effective targeting could be in the cell and how, by limiting the rise in ROS in a cell, the damage the cell takes from radiation can also be limited.

### 6.4. Carbonaceous Nanomaterials

One study synthesized ultrasmall BiOI quantum dots (BiOI QDs) surface-coated with Tween 20 to function as an intratumoral injection radiosensitizer [195]. The ultrasmall BiOI QDs surface-coated with Tween 20 were conjugated to have optimal tumor permeability and renal clearance for biocompatibility and practical use in cancer treatment (Table 7). The Bi(NO_3_)_3_·5H_2_O salt was introduced into a solution of water and ethanol mixture, followed by ultrasonication to create the quantum dots (QDs). Subsequently, a Tween 20 solution was employed to separate the BiOI QDs from the byproducts. HUVECs, 4T1 cells, and HeLa cells were tested under 6 Gy irradiation of X-rays to analyze the radiosensitization effect of the synthesized quantum dots. The ultrasmall BiOI QDs functioned as a radiosensitizer by facilitating the catalysis of the abundant hydrogen peroxide present in the tumor microenvironment into hydroxyl free radicals. The high concentrations of hydrogen peroxide are only overexpressed in cancer cells, so the quantum dots can achieve selective radiosensitization and enhance apoptosis of cancer cells. Furthermore, the synthesized quantum dots were shown to inhibit tumor cell proliferation, perturb the mitochondrial membrane potential, and inflict double-stranded DNA damage in cancer cells. As a result, the ultrasmall BiOI QDs surface-coated with Tween 20 showed great promise to complement radiotherapy by facilitating the formation of hydroxyl free radicals in the tested experimental systems. Another study formulated graphene quantum dots (GQDs) to function as a nano-radiosensitizer with enhanced uptake in tumor tissue to complement conventional tumor radiotherapy for more effective treatment of colorectal carcinoma [196]. The GQDs were synthesized with Hummer’s method and through photo-Fenton reactions of graphene oxide NPs. SW620 and HCT116 cells were tested under 3–6 Gy irradiation of gamma rays to determine the radiosensitization efficacy of GQDs. Researchers found that the synergy between the radiosensitization of the GQDs and ionizing radiation radiotherapy had the potential to upregulate G_2_/M cell cycle arrest, enhance apoptosis, decrease proliferation, and facilitate ROS production in colorectal carcinoma cells. Additionally, the GQDs and ionizing radiation synergy therapy were able to facilitate cell membrane blebbing, enhance chromatin agglutination, induce mitochondrial damage, condense cytoplasmic contents, and increase double-stranded DNA breaks in colorectal carcinoma cells. One group of researchers designed poly(lactic-co-glycolic acid) (PLGA) ultrasmall black phosphorous quantum dots (PQDs) for precise tumor radiosensitization to minimize the dose required for radiotherapy to be effective [197]. The QDs were made using the emulsion method, with PLGA NPs loaded with black PQDs. A375 and HeLa cancer cells, along with L02 normal cells, were treated with the PLGA ultrasmall black PQDs and 2 Gy X-ray irradiation to analyze the radiosensitization potential of the synthesized nanosystem. Results showed that the PLGA NPs function as carriers of the ultrasmall black PQDs to achieve controlled radiosensitization of tumors by mitigating off-target release and prolonging systemic circulation. The PLGA-ultrasmall black PQDs nanoparticulate system specifically targets the Arg-Gly-Asp-Gys sequence abundant in tumor tissue. In the acidic tumor microenvironment, the 2,3-dimethylmaleic acid anhydride shell decomposes, and the NPs achieve a positive charge, stimulating tumor cell uptake. Additionally, glutathione deoxidizes the disulfide bond of cysteine and enhances the release of the synthesized nanoparticle to increase tumor cell sensitivity to radiotherapy. Overall, comprehensive studies showed that the PLGA ultrasmall black PQDs facilitated apoptosis of cancer cells by promoting the formation of free radicals and exhibited optimal systemic biocompatibility for favorable applications in cancer therapy. Another group of researchers created black PQDs to complement the efficacy of conventional radiotherapy for applications in the treatment of aggressive renal cell carcinoma. The 786-O renal carcinoma cells and A498 cells were treated with black PQDs and 5 or 10 Gy of X-ray irradiation to determine their radiosensitization capabilities [198]. Studies showed that the black PQDs amplified ionizing radiotherapy-inducing DNA breaks through their interactions with the DNA-protein kinase catalytic subunit and the enhancement of its associated kinase activity. Additionally, the black PQDs inhibited the autophosphorylation of the DNA-protein kinase catalytic subunit at S2056, which is an essential site for DNA double-strand break repair. The black PQDs enhanced ionizing radiation-induced apoptosis in renal carcinoma cells, evidenced by increased DNA damage markers γH2AX and 53BP1. Animal experiments further validated the enhanced effectiveness of combining black PQDs with radiotherapy for treating renal cell carcinoma. One study synthesized GQDs doped with rare-earth up-conversion NPs to amplify the organelle-specific photodynamic effects of cancer therapy for clinical applications [199]. The 4T1 cells derived from the mouse mammary tumor cell line were treated with the conjugated QDs and UV irradiation, and the radiosensitization effects were assessed. Results showed that when the QDs were excited with UV rays, free radical formation was promoted. This directly led to a decrease in the mitochondrial membrane potential and stimulated irreversible tumor cell apoptosis. Comprehensive results indicated that the conjugated nanosystem addressed the limitations of conventional cancer radiotherapy and offered a feasible approach to organelle-specific precision to amplify tumor cell apoptosis. Another study formulated silver graphene quantum dots (AgGQDs) and assessed the combined therapeutic effects of silver graphene quantum dots, Resveratrol, and 2 Gy X-ray radiotherapy of HCT-116 colorectal cancer cells. Cell studies showed that the AgGQDs stimulated colorectal cancer cell apoptosis by inhibiting superoxide dismutase and glutathione peroxidase activities [186]. Additionally, the AgGQDs increase malondialdehyde concentrations, upregulated caspase-3 mRNA levels, and decreased cyclooxygenase 2 protein expression levels. Researchers concluded that the combination therapy of AgGQDs, resveratrol, and neoadjuvant radiotherapy significantly increased the apoptosis of colorectal cancer cells. One group of researchers manufactured iron–palladium-decorated single-walled carbon nanotubes (Fe-Pd-SWCNTs) and analyzed their potential to amplify free radical formation to supplement cancer radiotherapy [200]. The Fe-Pd-SWCNTs were prepared by chemical reduction and combined with 0–8 Gy X-ray irradiation to treat MCF-7 cells. These Fe-Pd-SWCNTs increased DNA double-stranded breaks by promoting the generation of ROS. ROS production was facilitated by the nanotubes’ ability to convert abundant hydrogen peroxide in the tumor microenvironment to hydroxyl radicals. At 200 µg/mL, the Fe-Pd-SWCNTs displayed ideal biocompatibility and the potential to serve as radiosensitizers for enhanced cancer therapy [200].

### 6.5. Cerium Oxide

Cerium oxide NP (CNP) is a rare earth metal that has many biomedical applications [201,202,203,204,205,206,207]. As a bulk material, cerium oxide relies on a fluorite structure that is pervious to defects depending on either the inherent stress state or the partial pressure of oxygen. This creates a material that works as a heterogenous catalysis with implications in the automotive, energy, optics, and electrical industries [208]. The specific mechanism is governed by the low activation threshold for lattice oxygen vacancies that enhance inherent oxidation processes. The gas-phase oxygen activation is not only facet-dependent but also intrinsic to surface sites. This relationship between lattice oxygen processes and surface processes creates the healing of vacant lattice oxygen sites that release gaseous oxygen [209]. The potential for oxygen storage release is relevant in the clinical setting as this material can alleviate hypoxic conditions. This process is further enhanced by manipulating the scale of synthesized cerium to NPs, which creates a higher surface defect ratio per cerium molecule. This enables nanoceria with its co-existence of Ce^3+^ and Ce^4+^ self-regenerative properties to sensitize tumor cells [210]. With respect to radiation incidences, the majority of research explains the radioprotective properties of CNP due to its ROS scavenging ability [211,212,213]. In fact, a recent article even explains the role of this radioprotective property in protecting astronauts from space radiation [19]. However, the oxygen storage capabilities of CNPs can enhance radio-sensitization effects by creating more reactants in the creation of radical oxygen species that can eliminate tumor cells (Table 7). For example, using a core–shell structure of scintillating NPs core with a CNP shell utilizes the catalase-like activity of ceria to decrease hypoxia within the cancer microenvironment. Computed tomography imaging of this novel hybrid nanomaterial also shows greater imaging potential (3.79 fold) than the clinical contrast agent of iohexol [214]. Additionally, CNPs can provide combinatorial effects by also delivering microRNA [16]. This application is useful as miR181a acts as a radiosensitizer through the regulation of the Chk2 pathway. By combining a 2D graphdiyne structure embedded with CeO_2_ NPs, hydrogen peroxide decomposition to oxygen enhances and reduces tumor hypoxia. The nanozyme is also subsequently able to protect the miRNA and enhance its therapeutic efficacy in radiation treatment (Figure 5). One of the most effective ways that studies have found for CNPs to increase cancerous cells’ sensitivity to radiation is through the use of a core–shell structure that is made up of cerium oxide particles. In a study performed using a shell–core radiosensitizer, the sensitizer was made of two layers of cerium oxide: CeO_2_ making up the core area, and the Ce_2_O_3_ making up the shell of the sensitizer [215]. The researchers performed in vitro studies using methyl orange and found that using UV-Vis radiation enabled the sensitizer to excite electrons and store them between the layers of cerium oxide (Table 8). The photo-excited electrons were trapped between the layers of cerium oxide and were then absorbed by oxygen molecules on the surface of the sensitizer, allowing for O_2_- radicals in nearby cells. These radical oxygen species would then induce oxidative damage upon tumorous cells, allowing for this radio-sensitizing compound to focus radiation in a collective way to sensitize cancer cells with future biomedical applications as well. Furthermore, recent studies have found that biofunctionalized nanoceria can achieve effective penetration across epithelial layers and controlled release of drugs. These properties render biofunctionalized nanoceria an attractive choice for synergistic cancer therapies encompassing radio sensitization and enhanced chemotherapeutic drug delivery [216].

## 7. Conclusions and Future Outlook

While nanotechnology is a robust field, and its application in medicine is promising, utilization in the clinical field is still in its infancy. However, its application in cancer treatment, especially as a cancer radiosensitizer, is leading the charge in federal approval and clinical adaptability. In fact, the first known FDA-approved NPs is PEGylated liposomal doxorubicin (known as Doxil), and it paved the way for nanodrug delivery and tumor targeting. The ability to passively target tumors and become available to tumor cells through the EPR effect shows how Doxil and other nanodrugs can be useful in cancer treatment [220]. This same nano-drug also presents excellent radiosensitivity that enables focused and targeted tumor cell radiolysis. However, according to FDA labels, Doxil is contraindicated with mediastinal irradiation. The risk of myocardial infarction is stated as the reasoning, but one clinical study shows that this is not necessarily the case [221]. Recent developments in intensity-modulated radiation therapy (IMRT) show reductions in cardiac toxicity of mediastinal irradiation. When combined with doxorubicin, the initial contraindications of the nanodrug are mitigated while improving overall patient outcomes than radiation alone [221]. Additionally, pegylated liposomal doxorubicin shows not only better bioavailability but also a reduction in cardiomyopathy. This sort of modification is important and not only vital to better patient outcomes but also to ensure FDA approval of the nanodrug [222]. This process of ensuring the clinical availability of the various nanomaterials discussed in this review will be more difficult with the utilization of inorganic compounds. Before inorganic NPs can be clinically implemented on a widespread scale, further extensive studies are required to elucidate their biocompatibility, their precise interactions with biological immune system mechanisms, and biological clearance processes [223].

It should also be discussed that the future of cancer treatment will drastically change with the advent of machine-learning algorithms and artificial intelligence. Monumental medical discoveries, such as using machine learning to screen antibiotic molecules to target A.baumannii, are merely the surface of what algorithmic processing can do [156]. The role of material science and nanotechnology in this emerging new reality is significant, especially with the discovery of new materials, prediction of function, and optimization of synthesis. The growing plethora of material science data and references will be crucial in uncovering new developments in the realm of radio-sensitizing nanomaterials [224].

Due to the plethora of cancer variations and the necessity for individualized approaches, there is a high demand for a streamlined approach to treating cancers. We expect to see cancer only increasing in prevalence among scholarly articles, with more novel strategies to enhance cancer treatment outcomes in question. The growing body of scientific knowledge related to cancer and its potential treatment strategies is encouraging. The introduction of nanotechnology into medicine has opened a new vein of possibility when it comes to targeted treatments for countless cancer types. Our increasing ability to strike a chemical balance between the radioprotection of healthy cells and radio sensitization of cancer cells has significant potentiality for the advancement of cancer therapeutics overall. There is a wide range of delivery methods, as well as options of particle type and characteristics that may produce individualized desirable treatment outcomes. Future research will likely delve into detail of what nanosystems, elements, and morphologies are most effective for specific purposes. Overall, nanotechnology poses a propitious addition to the field of cancer research and the ambition for better treatment outcomes.

## Figures and Tables

**Table 1:** Summary of commonly prescribed chemotherapeutic drugs and their corresponding mechanism of action.

Mechanism of Action	Common Drugs
Alkylating Agents: This class of drugs reacts with nucleophilic sites on nucleic acids and proteins to stimulate the formation of unstable alkyl groups. This reaction then inhibits DNA replication and transcription.	Bendamustine, cyclophosphamide, ifosfamide, carmustine, lomustine, temozolomide, carboplatin, thiotepa, cisplatin, oxaliplatin, busulfan, dacarbazine, procarbazine.
Antimetabolites: Interfere with DNA methyltransferase and/or DNA polymerase to inhibit DNA replication.	Cytidine analogs: cytarabine; azacitidine; gemcitabine; decitabine. Folate antagonists: methotrexate; pemetrexed. Purine analogs: cladribine; clofarabine; nelarabine. Pyrimidine analogs: fluorouracil; capecitabine.
Antimicrotubular Agents: Topoisomerase Inhibitors: Inhibit topoisomerase 1 or topoisomerase 2 to inhibit DNA repair and block DNA and RNA synthesis. Taxanes: Disruption of microtubule assembly, thereby inhibiting cell cycle progression in the M phase. Vinca alkaloids: Bind to tubulin to inhibit microtubule development. This complex then causes cell cycle arrest in metaphase.	Topoisomerase 1 Inhibitors: irinotecan; topotecan. Topoisomerase 2 Inhibitors: doxorubicin; Daunorubicin; idarubicin; mitoxantrone. Taxanes: paclitaxel; docetaxel; cabazitaxel. Vinca alkaloids: vinblastine; vincristine; vinorelbine.
Antibiotics: Inhibit the synthesis of RNA and DNA Binds to DNA to produce single and double-stranded breaks in DNA.	Actinomycin D, bleomycin, daunomycin.
Inhibits ribonucleoside diphosphate reductase, thus cell cycle progression in the S phase.	Hydroxyurea.
Targets RAR-alpha pathway, thereby promoting cell differentiation.	Tretinoin.
Stimulates cell differentiation.	Arsenic trioxide.
Inhibits the functions of proteasomes.	Bortezomib.

**Table 2:** Summary of different energetic rays used in various types of cancer treatment.

Radiation Type	Methodology	Subjects	Results	Why	Source
X-rays	Metal–organic frameworks were injected into local tumors, then treated with X-ray radiation.	Mouse models of breast and colorectal cancer.	Low-level X-ray radiation was able to remove the local tumors and subsequently prevent tumors from reoccurrence.	The frameworks coupled with X-ray radiation helped to overcome the limitations of cytotoxic T-cell response, and X-ray radiation served to perform an “in situ vaccination”.	[110]
X-rays	X-rays are used to induce a photodynamic therapy process, which is coupled with radiation therapy to produce significantly better tumor-killing abilities.	In vitro: H1299-Luc cells In vivo: 5–6 week athymic nude mice	The development of a treatment known as X-PDT demonstrated increased efficiency both in vivo and in vitro against tumorous cells, especially more thermodynamically resistant cells.	X-PDT was shown to increase the penetrance of light, allowing for a PDT process to occur while combining it with radiotherapy.	[111]
Gamma rays	Gamma Knife surgery allows for precise treatments to occur, most commonly found in the brain.	A total of 1475 patients afflicted with acoustic neurinomas.	The study showed that among the 1475 patients who underwent Gamma Knife Surgery, only 8% had enlargement afterward over a 3-year period. Compared with microsurgery, Gamma Knife also allows for a lower morbidity rate and fewer complications.	Gamma Knife suppresses tumor growth and provides tumor control by breaking the ability for the tumor to reform and grow through its DNA damage.	[112]
Gamma Rays	Gamma rays used through prompt exposure were found to leave nearly the same amount of radiation in soft tissue as compared to the lower energy waves that were used.	Results obtained from statistical analysis	The study found that gamma rays differed by a factor of two in their dosing when used in a therapeutic setting and allowed for the necessary increase in energy when dealing with thermoresistant cells in cancer treatment.	Gamma rays produce significantly more energy than X-ray radiation and can be modified to provide a therapeutic treatment.	[113]
Electron Beam	Patients underwent an external beam treatment with dosages of up to 45 gy.	37 patients with soft tissue sarcoma	The study found that among those who underwent electron beam treatment, nearly 83% remained sarcoma-free, whereas that percentage dropped to 59% for those who did not. Additionally, patients provided excellent local control and a disease-free state at acceptable mortality.	The treatment allowed for the removal of the sarcoma from the soft tissue and allowed for the area to remain cancer-free.	[114]
Electron Beam	Intraoperative electron beam radiation therapy (IORT).	Sixty-five patients with recurrent areas or high-risk areas of cancer.	The study showed that the 5-year rate of survivability and disease-free rate increased from 60 and 32%, respectively, to almost 88% and 53% with the undergoing of IORT.	IORT functions to help the complete recitation of dangerous or recurrent tumors.	[115]

**Table 3:** Summary of recent studies evaluating the efficacy of integrating nanomaterials with ionizing radiation for enhanced diagnostic and therapeutic applications in cancer.

Type	Application	Radiation Used	Mechanism	Synthesis	Size	Ref.
Liposomes functionalized with gold nanoclusters	Imaging and Diagnostic	Wide variety	Allows for colorimetric detection of HER-2-positive breast cancer cells	Extrusion Method	175.04 +/− 2.45 nm	[142]
AuNPs and Liposomes	Therapeutic	Ultraviolet (UV) radiation	DNA-directed assembly of biomolecules for alleviation of pathogenic symptoms of various diseases	Extrusion Method	103 nm	[143]
AuNPs	Imaging	Photoluminescence	Allows for detection and imaging of intracellular thiols that can be significant biomarkers for chronic diseases and their progression	Not Specified	1.8–3.0 nm	[144]
Titanium Dioxide	Therapeutic	UV radiation, X-ray radiation (when titanium dioxide NPs were functionalized with gadolinium)	Amplify the formation of reactive oxygen species (ROS) in corresponding tumor tissue to facilitate apoptosis for cancer therapy (i.e., glioblastoma)	Not Specified	Not Specified	[145]
Quantum Dots synthesized from CaF, LaF, ZnS, or ZnO	Therapeutic	Light waves (most biocompatible), X-rays, gamma rays	Generation of radicals upon light radiation for cancer therapy	Not Specified	Not Specified	
Superparamagnetic Iron Oxide	Therapeutic	X-rays	Exhibit cytotoxic effects on cancer cell lines through the facilitation of radicals’ production	Not Specified	Not Specified	[146]
Polymer	Therapeutic	^ 60 ^ Co source emitting gamma rays	Function as an Amifostine carrier and radiosensitizer to allow cancer cells to amplify the effects of radiation therapy (part of synergistic therapy)	Not Specified	Not Specified	[147]
Solid Lipid NPs	Therapeutic	N/A	Function as a radiosensitizer and deliver small interfering RNAs (siRNAs) antagonists for programmed cell death ligand-1 (PD-L1) and epidermal growth factor receptor (EGFR)	Melt–Emulsification	51.3 nm	[148]
AuNPs	Therapeutic	Photon Beam	Function as a synergistic radiation therapy to amplify mitosis perturbation pathways	Not Specified	Not Specified	[149]
AuNPs	Therapeutic	Photon Beam	Control the release and dosimetry of radiotherapy in cancer cells (synergy therapy)	Citrate-Reduction Technique	20.90 +/− 0.14 nm	[150]

**Table 5:** Summary of studies discussing the utilization of AgNPs to enhance the radiosensitization effect of conventional radiotherapy for improved cancer outcomes.

Nanoparticle and Functionalization	Synthesis Method and Size	Experimental System	Radiation Utilized and Dose	Mechanism	Notable Results	Ref.
AgNPs Functionalized with Polyethylene Glycol and Aptamer As1411	Electrochemical Synthesis, 18 nm.	C6 glioma cells, human microvascular endothelial cells.	X-Rays, 6 MV, 200 cGy/min.	Dark-field microscopy and confocal laser scanning microscopy were utilized to evaluate the targeting properties of the synthesized NPs MTT and Annexin V-FITC/PI assays and C6 glioma spheroid models were utilized to assess tumor spheroid penetration and apoptotic effects.	PEG and As1411 surface modifications significantly enhanced Ag nanoparticle uptake in glioma tumor cells. The synthesized NPs successfully penetrated the core of tumor spheroids. The AgNPs modified with PEG and As1411 facilitated amplified apoptosis rates. Synergistic therapy of PEG-As1411 AgNPs and X-ray irradiation prolonged survival time of C6 glioma mice.	[180]
PEGylated graphene quantum dot-decorated Silver Nanoprisms	Synthesized by exploiting the non-covalent electrostatic interactions created between the AgNPs and the PEG-graphene quantum dots Size: 18–45 nm.	In vitro: HCT 116 and HT29 (relatively radiation-resistant) colorectal cancer cells; both cell lines are derived from humans. In vivo: Male Swiss nu/nu mice (age: 5–8 weeks).	X-rays, 2–10 Gy.	The PEGylated AgNPs did not significantly amplify ROS production in irradiated HCT 116 but increased ROS production by 18% in irradiated HT29 cells. The NPs were able to pose radiosensitive effects on colorectal cancer cells by amplifying ROS production pathways.	The combination therapy of conventional radiation and the administration of the synthesized NPs stunted colorectal tumor growth and prolonged the survival time when compared to groups treated with only radiotherapy.	[181]
AgNPs functionalized with anti-EGFR antibodies	Thermal Reduction Method Size: 20 nm.	In vitro: Human Nasopharyngeal carcinoma epithelial cells. In vivo: Mouse model with nasopharyngeal carcinoma.	X-rays 0, 2, 4, 6, or 8 Gy	The AgNPs functionalized with anti-EGFR antibodies posed radiosensitive effects by downregulating the expression of the mitosis arrest proteins, Rad51, Ku-80, and Ku-70, in human nasopharyngeal carcinoma epithelial cells. This downregulation facilitated apoptosis in the cancer cells.	The Ag-anti EGFR NPs inhibited the proliferation of nasopharyngeal carcinoma epithelial cells and stimulated their apoptosis via G2 cell cycle arrest.	[182]
AgNPs in Conjunction with Cisplatin	AgNPs were capped with polyvinyl pyrrolidone. Size: 23.1 +/− 6.9 nm.	In vitro: Ovarian cancer cell lines (SKOV3, A2780, and OVCAR3).	Not Specified.	AgNPs facilitated radio sensitization by amplifying ROS production pathways and inducing DNA damage. A2780 cells treated with Ag NPs (100 µg/mL) became rounded and enhanced the loss of adherent ovarian cancer cells. A secondary mechanism in which the Ag NPs facilitated apoptosis was by decreasing the glutathione/homodimer disulfide ratio.	The AgNPs inflicted cytotoxic effects via DNA damage and ROS amplification on the A2780 and SKOV3 cell lines. The OVCAR3 cell lines were not as responsive to the AgNPs. Overall, the combination therapy of cisplatin and AgNPs displayed synergistic effects and resulted in a favorable cisplatin dose reduction in the treatment of ovarian cancer cells.	[183]
AgNPs	Electrochemical Synthesis Method Size: 26.87 nm.	U251 and C6 glioma cell lines.	X-rays, 6 MeV Dose Rate: 200 cGy/min.	AgNPs facilitate therapeutic effects in cancer by stimulating apoptosis and facilitating destructive autophagy through manipulation of 3-methyladenine Hypoxic U251 glioma cell lines treated with X-rays and Ag NPs exhibited significantly lower mitochondrial membrane potential when compared to hypoxic U251 glioma cell lines treated with X-ray irradiation alone. A decrease in mitochondrial membrane potential is an early step in the apoptosis signaling cascade.	The combination therapy of AgNPs coupled with radiotherapy elicited significant anti-glioma effects in hypoxic C6 and U251 glioma cell lines.	[184]
Resveratrol-loaded AgNPs Conjugated with Graphene Quantum Dots	Not Specified.	HCT-116 colorectal cancer cells.	X-rays 2 Gy.	The resveratrol-loaded AgNPs conjugated with graphene quantum dots promoted radio sensitization in HCT-116 colorectal cancer cells by reducing superoxide dismutase (SOD) and glutathione peroxidase (GPX) enzyme activities while increasing malondialdehyde (MDA) levels. Additionally, the NPs facilitated apoptosis by upregulating caspase-3 mRNA expression and decreasing cyclooxygenase (COX-2) protein expression.	Colorectal cancer cell viability was significantly hindered, and apoptosis was amplified when treated with X-ray irradiation and silver quantum dot-Resveratrol NPs The synergistic treatment upregulated caspase-3 mRNA and decreased COX-2 protein expression in colorectal cancer cells.	[185]
AgNPs	Rapid Extracellular Biosynthesis by using the fungus Penicillium aurantiogresium. Size: 12.7 nm.	MCF-7 and MCT breast cancer cell lines.	Gamma rays 6 Gy Dose Rate: 0.675 Gy/s.	The Ag NPs complemented gamma-ray irradiation treatment by promoting apoptosis through activation of lactate dehydrogenase, downregulation of Bcl-2 genes upregulation of caspase-3.	AgNPs displayed dose-dependent cytotoxicity and confirmed their capability to function as a potent radiosensitizer for gamma irradiation. Additionally, administration of silver NPs altered cell morphology, inhibited cell proliferation, activated lactate dehydrogenase, and caspase-3. The activation of lactate dehydrogenase and caspase-3 and the downregulation of Bcl-2 genes induced apoptosis.	[186]
AgNPs Synthesized with normoxic polyacrylamide gelatin and tetrakis hydroxy methyl phosphonium chloride polymer gel	The AgNPs were synthesized by utilizing laser ablation. Silver NPs were embedded in the final step of the polymer gel preparation process. Size: 20 nm.	Not Applicable.	Gamma rays 6–25 Gy.	Different volumes of AgNPs were experimented with to find the ideal configuration for applications in dose enhancement. At an ideal dose, the AgNPs facilitated polymerization of the polymer gel, which thereby increased the received dose of anti-cancer drugs.	The dose response of anti-cancer drug accumulated in tumor tissue increased by 11.82% when AgNPs were implemented at a concentration of 2 mL. In the presence of AgNPs, the maximum penetration dose of drugs observed was 0.5 cm. Overall, the integration of silver NPs in the system increased the optical density of the drug delivery fabrication.	[187]
AgNPs Functionalized with Carbon Nanodots and Polyethylene Glycol	Synthesis Method is not specified. Size: 5–100 nm.	In vitro: Du145 prostate cancer cells.	X-rays Not Specified	PEG was utilized to improve the NPs’ affinity to accumulate within prostate cancer cell tissue. Carbon nanodots were incorporated within the Ag nanostructure to improve stability and radio-sensitizing effects. The fabricated NPs were thought to induce radiosensitization by modulating DNA damage and inducing hyperthermia.	The combination therapy of radiation and Ag NPs provided a synergistic effect and reduced prostate cancer cell survival by 50%.	[188]
Bimetallic Au/AgNPs	Bile acid molecules were used to synthesize the branched bimetallic Au/AgNPs. The branched structure of the nanoparticulate system optimized surface area and led to an enhancement in catalytic activity. Size: Not Specified.	In vitro: N1-S1 rodent hepatocellular carcinoma cells	External Beam Radiation, 90Y Thera Sphere exposure. 0–10 Gy.	The bimetallic gold/silver nanoparticulate system functioned as radiosensitizer by facilitating the generation of reactive oxygen species. This process occurred by gold/silver-mediated transfer of electrons to intracellular oxygen.	Exposure to external beam radiation or 90Y Thera Sphere and administration of the bimetallic Au/AgNPs caused significant increases in ROS production. The ROS generation induced oxidative stress and facilitated apoptosis in hepatocellular carcinoma cells.	[189]

**Table 6:** Summary of SiNPs used for radio sensitization during the cancer treatment.

Nanoparticle and Functionalization and Composition	Synthesis Method and Size	Experimental System	Radiation Utilized and Dose	Mechanism	Notable Results	Ref.
Hyperbranched polyamidoamine grafted onto the surface of amorphous SiNPs	Hyper-branched PAMAM was grafted onto the surface of synthetic amorphous NPs; 40 mg was dissolved in a water/ethanol mixture; the particles were conjugated in fluorescent dye; 20–50 nm sized particles were yielded.	In vitro SK-BR3 Breast cancer cells.	X-ray; 8 Gy.	SiNPs were internalized by the breast cancer cells, allowing for stronger local control. Internalized SiNPs helped to disturb the permeability of the lysosomal membrane, leading to disruptions that can cause apoptosis or cellular necrosis on a larger scale.	Radiation reduced the presence of breast cancer cells when it was applied with the SiNPs, showing that silica could be used to potentially help normal cells during cancer treatment.	[190]
Mesoporous SiNPs	MSN-benzimidazole was prepared using a previous method. The particles were dissolved in a valproic acid solution and then centrifuged. No specified particle size.	In vitro Rat glioma C6 and human glioma U87.	X-ray and IR; 4 and 8 Gy	Recognizes the over-producing folic acid in cancerous cells. The NPs then, in turn, release VPA into the environment. Silica is used to do targeted drug delivery and controlled release.	Targeted cancer cells reduced the effects of radiation and also showed a higher rate of cell death and enhanced inhibition. A lower radiation dose may be required to kill cancer cells.	[191]
Uncapped and aminosilanized SiNPs	Reverse-micelle wet-chemistry procedure. No specified size.	MCF tumor cells and 3T3 tissue cells.	X-rays; 3 Gy	The synthesized NPs punctured into the cellular mitochondria, creating ROS in the cellular body and provoking oxidative stress.	Increased ROS concentrations in the tumorous cells by almost 180%, whereas only changing the tissue by 120%. Results show that cytotoxicity favors the tumor cells.	[192]
Gold-Nanorod-Filled Mesoporous Silica Nanobeads	Formulate a synergistic therapy by combining conventional radiotherapy with gold-nanorod-filled mesoporous silica nanobeads to treat oral squamous carcinoma.	Organic Template Method 120–160 nm CAL-27 and L929 cells.	X-ray 2 Gy	Synthesis procedures included the inclusion of cetyltrimethylammonium bromide (CTAB) in the nanostructure to avoid aggregation and agglomeration.	Effective cellular uptake of the synthesized NPs was achieved in CAL-27 cells. Compared to groups treated only with irradiation, groups treated with the synergistic therapy of radiotherapy and Mesoporous Silica Nanobeads had significantly higher incidences of ROS formation.	[193]
Gadolinium Mesoporous Silica Nanoparticles (Gd-MSNPs)	Develop a novel and more effective radiotherapeutic for the eradication of tumor masses.	Sol–Gel method 139 nm OVCAR8 human ovarian cancer cells; HEK293 human embryonic kidney cells.	X-rays 50.25 keV.	The surface of the Gd-MSNPs was functionalized with amine groups to ensure the loading of gadopentetic acid onto the Gd-MSNs	In vitro studies indicated that incubation of the cancer cells with 50 ng of the synthesized NPs completely disintegrated the tumor spheroids.	[194]

**Table 7:** Summary of studies determining the radiosensitization potential for carbonaceous nanomaterials to function as a supplemental therapy to conventional radiotherapy for improved cancer prognosis.

Nanoparticle and Functionalization	Objective	Radiation Utilized and Dose	Experimental System	Synthesis Method	Size	Mechanism	Notable Results	Ref.
Ultrasmall BiOI QDs Surface-Coated with Tween 20	Develop a nanomaterial-based intratumoral injection radiosensitizer with optimal tumor permeability and renal clearance for biocompatible and practical use in cancer treatment.	X-ray 6 Gy	HUVECs, 4T1 cells, and HeLa cells	KI was dissolved in a solution mixture of DI water and ethanol. Ultrasonication was performed, and Bi(NO_3_)_3_·5H_2_O was added to the solution. After this, centrifugation was performed, and the supernatant was injected into Tween 20 solution. Centrifugation was performed again, and ultrafiltration was used to process the supernatant. The resulting supernatant solution was the BiOI QDs.	3 nm	The ultrasmall BiOI QDs function as a radiosensitizer by facilitating the catalysis of the abundant hydrogen peroxide present in the tumor microenvironment into hydroxyl free radicals.	The ultrasmall BiOI QDs surface-coated with Tween 20 are biocompatible and mitigate toxicity attributed to long-term retention through systemic elimination via renal metabolic clearance pathways. The synthesized ultrasmall BiOI QDs surface-coated with Tween 20 showed great promise to complement radiotherapy by facilitating the formation of hydroxyl free radicals in the tested experimental systems.	[196]
GQDs	Synthesize a nano-radiosensitizer with enhanced uptake in tumor tissue to complement conventional tumor radiotherapy for more effective treatment of colorectal carcinoma.	Gamma rays 3–6 Gy	SW620 and HCT116 cells	Hummer’s method. The graphene quantum dots were synthesized through the photo-Fenton reaction of graphene oxide NPs.	18 nm	The synergy between the radiosensitization of the GQDs and IR radiotherapy have the potential to upregulate G2/M cell cycle arrest, enhance apoptosis, decrease cell proliferation, and facilitate ROS production in colorectal carcinoma cells.	The GQDs showed great promise in enhancing the sensitivity of colorectal carcinoma cells to IR therapy. The GQDs and IR synergy therapy were able to facilitate cell membrane blebbing, enhance the agglutination of chromatin, induce mitochondrial damage, condense cytoplasmic contents, and increase double-stranded DNA breaks in colorectal carcinoma cells.	[197]
Poly(lactic-co-glycolic acid) (PLGA) Ultrasmall Black PQDs	Design a nano-system for precise tumor radiosensitization to minimize the dose required for radiotherapy to be effective.	X-rays 2 Gy	A375 cancer cells, HeLa cancer cells, L02 normal cells.	Emulsion Evaporation Method. PLGA NPs are loaded with black PQDs to create the PLGA-ultrasmall black PQDs.	150 nm	The PLGA NPs function as carriers of the ultrasmall black PQDs to achieve controlled radiosensitization of tumors by mitigating off-target release and prolonging systemic circulation. The PLGA-ultrasmall black phosphorous quantum dot nanoparticulate system targets the Arg-Gly-Asp-Gys sequence abundant in tumor tissue. In the acidic tumor microenvironment, the 2,3-dimethylmaleic acid anhydride shell decomposes, and the NPs acquire a positive charge, stimulating tumor cell uptake. Additionally, glutathione deoxidizes the disulfide bond of cysteine and enhances the release of the synthesized NP to increase tumor cell sensitivity to radiotherapy.	The synthesized nanosystem facilitated apoptosis of cancer cells by promoting the formation of free radicals and exhibited optimal systemic biocompatibility.	[198]
Black PQDs	Synthesize black PQDs to complement the efficacy of conventional radiotherapy for applications in treatment of aggressive renal cell carcinoma.	X-rays 5 or 10 Gy.	786-O Renal Carcinoma Cells, A498.	1 g of black PQDs was immersed within N-methyl-2-pyrrolidinone for 12 h at 140 degrees Celsius.	10 nm	The black PQDs amplify ionizing radiotherapy, inducing double-stranded DNA breaks through their interactions with DNA-protein kinase catalytic subunit and the enhancement of its kinase activity. Additionally, the black PQDs inhibit the autophosphorylation of the DNA-protein kinase catalytic subunit at S2056, which is an essential site for DNA double-strand break repair.	Overall, the black PQDs amplify ionizing radiation-induced apoptosis of renal carcinoma cells, as confirmed by the upregulation of DNA damage biomarkers γH2AX and 53 BP1. Animal experiments confirmed that the synergistic therapy of radiosensitizing black PQDs and ionizing radiotherapy have enhanced efficacy for treating renal cell carcinoma.	[199]
GQDs doped with rare-earth upconversion NPs	Design a novel radiosensitization nanosystem to amplify the organelle-specific photodynamic effects of cancer therapy for clinical applications.	UV rays not Specified.	4T1 cells derived from the mouse mammary tumor cell line.	Not Specified.	100 nm	When excited with UV rays, the GQDs doped with rare-earth NPs promote the formation of radicals that facilitate the decrease in mitochondrial membrane potential. This process then stimulates irreversible tumor cell apoptosis.	The conjugated nanosystem addresses the limitations of conventional cancer therapy and offers a feasible approach to organelle-specific precision to amplify tumor cell apoptosis.	[200]
AgGQDs	Assess the combined therapeutic effects of silver graphene quantum dots, Resveratrol, and radiotherapy on HCT-116 colorectal cancer cells.	X-rays 2 Gy	HCT-116 colorectal cancer cells.	Not Specified.	Not Specified	The AgGQDs stimulated colorectal cancer cell apoptosis by inhibiting superoxide dismutase and glutathione peroxidase activities. Additionally, the AgGQDs increased malondialdehyde concentrations, upregulated caspase-3 mRNA levels, and decreased cyclooxygenase 2 protein expression levels.	The combination therapy of AgGQDs, Resveratrol, and neoadjuvant radiotherapy significantly increased apoptosis of colorectal cancer cells.	[186]
Iron–Palladium-Decorated Single-Walled Carbon Nanotubes	Analyze the potential for iron–palladium-decorated single-walled carbon nanotubes to amplify free radical formation to supplement radiotherapy for the treatment of cancer.	X-rays 0, 2, 4, 6, 8 Gy.	MCF-7 cells.	Chemical Reduction Method.	3–4 nm	The Fe-Pd carbon nanotubes enhanced the frequency of DNA double-stranded breaks by facilitating the generation of reactive oxygen species (ROS). ROS was generated by converting the abundant hydrogen peroxide levels in the tumor microenvironment to hydroxyl radicals.	The Fe-Pd carbon nanotubes at 200 µg/mL had optimal biocompatibility and demonstrated the potential to function as radiosensitizers for improved cancer therapy.	[201]

**Table 8:** Summary of studies analyzing the ability of CNPs to promote radiosensitization effects in cancer cells.

Nanoparticle and Functionalization and Composition	Synthesis Method and Size	Experimental System	Radiation Utilized and Dose	Mechanism	Notable Results	Ref.
CNPs	Core–Shell structure.	In vitro Methyl Orange.	UV-Vis radiation.	Light absorption by the shell structure allows for the formation of defects and photo-excited electrons. These electrons, once excited, are trapped between the CeO_2_ and the Ce_2_O_3_. These electrons are captured by oxygen at the surface, forming O^2−^ radicals.	Degradation was measured to be around 1.5–2.7 times better when the structure was exposed to UV-VIS radiation	[215]
Cerium Oxide SCNPs@DMSN@CeOx-PEG	Multifunctional core–shell radiosensitizer.	In vitro (in situ experiment). Measurement as a blue-shift scale.	X-rays.	Core-radiosensitizer absorbs high-energy X-rays, resulting in the formation of a photoinduced electron hole to generate reactive oxygen species.	Use of the radiosensitizer is about 3.79 times better than the use of iohexol (a clinical contrast agent).	[214]
GDY-Cerium Oxide nanocomposites	Novel 2D graphdiyene can anchor and disperse CNPs.	In vitro use of radioresistant cell models to determine capabilities of a nanostructure GDY. In vivo study of the distribution of nanostructure in mice with subcutaneous tumors.	Multistage radiation therapy.	GDY is a 2D carbon material that enables unique drug loading as well as photothermal ability.	Nanozymes with the GDY-CeO_2_ complex are found to provide increased CAT-mimetic behavior as well as effective radio sensitization for ESCC. Also found to be nontoxic for cells and well dispersed throughout live animal models.	[217]
CNPs Metal–Organic Frameworks	Synthesized through the one-step method in which ultrasmall homogenous cerium oxide particles are isolated.	In vitro blocked energy supply effects in cells.	None listed.	CNPs have catalytic hydrolysis on ATP and can be utilized to treat synergistic tumors with little to no side effects.	OXD-like and ATP deprivation allowed for high oxidative damage and blockage of energy supply effects.	[218]
Amine-modified CNPs	Conjugated with neogambogic acid.	In vitro MCF-7 breast cancer cells in G2/M phase.	X-rays 5-Gy radiation therapy (30 Gy total).	Extreme anticancer behavior was found using different mechanisms. Autophagy was induced by killing the cancer cells.	Surface modification of CNPs additionally decreased radiation-induced ROS formation.	[219]

## Data Availability

Not applicable.
